# Studying Salt-Induced Shifts in Gene Expression Patterns of Glucosinolate Transporters and Glucosinolate Accumulation in Two Contrasting *Brassica* Species

**DOI:** 10.3390/metabo14040179

**Published:** 2024-03-22

**Authors:** Samia Fatima, Muhammad Omar Khan, Nadia Iqbal, Muhammad Mudassar Iqbal, Huma Qamar, Muhammad Imtiaz, Penny Hundleby, Zhengyi Wei, Niaz Ahmad

**Affiliations:** 1 National Institute for Biotechnology and Genetic Engineering College (NIBGE-C), Pakistan Institute for Engineering and Applied Sciences (PIEAS), Faisalabad 38000, Pakistan; saminafatime@nibge.org (S.F.); muhammadkhan@nibge.org (M.O.K.); nadiaiqba@nibge.org (N.I.); muhammadiqbal@nibge.org (M.M.I.); m.imtiazpk92@nibge.org (M.I.); 2 Oilseeds Research Institute, Ayub Agricultural Research Institute, Faisalabad 38000, Pakistan; huma.qamar@research.uwa.edu.au; 3 School of Biological Sciences, The University of Western Australia, Crawley, WA 6009, Australia; 4 Department of Crop Genetics, John Innes Centre, Norwich Research Park, Norwich NR4 7UH, UK; penny.hundleby@jic.ac.uk; 5 Maize Research Institute, Guangxi Academy of Agricultural Sciences, Nanning 530007, China

**Keywords:** glucosinolate transporters, glucosinolates, gene expression, *Brassica*, salt tolerance, antioxidants

## Abstract

*Brassica* crops are well known for the accumulation of glucosinolates—secondary metabolites crucial for plants’ adaptation to various stresses. Glucosinolates also functioning as defence compounds pose challenges to food quality due to their goitrogenic properties. Their disruption leaves plants susceptible to insect pests and diseases. Hence, a targeted reduction in seed glucosinolate content is of paramount importance to increase food acceptance. *GLUCOSINOLATE TRANSPORTERS* (*GTRs*) present a promising avenue for selectively reducing glucosinolate concentrations in seeds while preserving biosynthesis elsewhere. In this study, 54 putative GTR protein sequences found in *Brassica* were retrieved, employing Arabidopsis GTR1 and GTR2 templates. Comprehensive bioinformatics analyses, encompassing gene structure organization, domain analysis, motif assessments, promoter analysis, and cis-regulatory elements, affirmed the existence of transporter domains and stress-related regulatory elements. Phylogenetic analysis revealed patterns of conservation and divergence across species. Glucosinolates have been shown to increase under stress conditions, indicating a potential role in stress response. To elucidate the role of GTRs in glucosinolate transportation under NaCl stress in two distinct *Brassica* species, *B. juncea* and *B. napus*, plants were subjected to 0, 100, or 200 mM NaCl. Based on the literature, key *GTR* genes were chosen and their expression across various plant parts was assessed. Both species displayed divergent trends in their biochemical profiles as well as glucosinolate contents under elevated salt stress conditions. Statistical modelling identified significant contributors to glucosinolate variations, guiding the development of targeted breeding strategies for low-glucosinolate varieties. Notably, *GTR2A2* exhibited pronounced expressions in stems, contributing approximately 52% to glucosinolate content variance, while *GTR2B1/C2* displayed significant expression in flowers. Additionally, *GTR2A1* and *GTR1A2/B1* demonstrated noteworthy expression in roots. This study enhances our understanding of glucosinolate regulation under stress conditions, offering avenues to improve *Brassica* crop quality and resilience.

## 1. Introduction

The *Brassicaceae* family of genus *Brassica* encompasses diverse plant species, including vegetables, oilseeds, condiments, and ornamentals [1,2]. Among these, the *Brassica* complex, which consists of six crucially important crops, holds global significance in agriculture, food, and industry [3]. This complex, also known as U’s triangle, comprises three diploid species (*Brassica compestris*, *B. nigra*, *B. oleracea*) and three allotetraploid species (*B. napus*, *B. juncea* and *B. carinata*) [4]. *B. napus* and *B. juncea*, commonly known as rapeseed and mustard, are prominent oilseed crops with high seed oil contents (40–48%), ranking as the second most crucial oilseed crops after soybean [5]. 

In many countries like India and Pakistan, rapeseed and mustard are the main oilseed crops, mainly cultivated in marginal lands due to various factors, including competition with the major cereal crop, e.g., wheat, non-availability of high-yielding varieties, lack of incentives, etc. The crops are grown on marginal saline lands [6]. Salinity is a significant stressor that affects crop yields worldwide, affecting 20% of cultivated and 33% of irrigated lands globally [7]. *Brassica* crops are susceptible to salinity stress at all growth stages, leading to yield losses and quality deterioration in terms of increased erucic acid and glucosinolates in seed oil and meal [8,9,10]. Total glucosinolate content has also been found to be increased under salt stress due to increased secondary metabolism [11,12].

Glucosinolates are nitrogen- and sulfur-containing secondary metabolites exclusive to the family *Brassicaceae* [13]. They have also been found in several other families of order *Brassicales* other than *Brassicaceae*, including *Capparaceae* (Capers), *Caricaceae* (papaya). and *Moringaceae* (Moringa) [14,15,16]. Glucosinolates, as constitutive components of cells, are synthesized early in the plant’s life cycle and are stored in either vacuoles or specialized idioblasts. The biological activity is attributed to hydrolysis products resulting from the breakdown of glucosinolates by the myrosinase enzyme upon damage [17]. These breakdown products exhibit a range of activities such as fungicidal, antimicrobial, and cancer-preventing, antigoitrogenic and anti-inflammatory [18,19,20]. Some glucosinolates and their degradation products, such as progoitrin and epi-progoitrin, can reduce palatability [21]; others, like nitriles, may impair liver and kidney function [22], while thiocyanates can make iodine unavailable [23] and 5-vinyl-1,3-oxyzolodine-2-thione can affect thyroid morphology as well as physiology. In animals, consuming glucosinolate-rich meals above the acceptable limit (30 µmol/g of oil-free seed meal) can result in goitrogenic effects [24,25,26]. Additionally, the pungent smell of sinigrin or allyl glucosinolate can make it undesirable for animals [27]. 

Glucosinolates are crucial in plant defence against insects and pathogens [28]. They deter feeding and inhibit the growth of herbivores, including birds, slugs, and insects [29]. When plants are under attack, they increase their glucosinolate levels through the de novo synthesis of glucosinolates and transport of glucosinolates from other plant parts [30,31,32,33]. Glucosinolate hydrolysis products, particularly isothiocyanates, exhibit antimicrobial and fungicidal activities against bacterial and fungal pathogens [34,35,36]. In cabbage, resistant varieties have been found to increase their aliphatic and indolic glucosinolate content and the gene expression involved in their biosynthesis when attacked by *Sclerotinia sclerotiorum* [37]. Glucosinolates have also been reported to be involved in defence mechanisms against abiotic stresses by activating signalling pathways like jasmonic acid (JA) and salicylic acid (SA) pathways [38], inferring the role of these secondary metabolites in plant defence mechanisms. Disruption of glucosinolates in *Brassica* plants made them susceptible to disease and insect attack [39]. 

*GLUCOSINOLATE TRANSPORTERS* (*GTRs*) represent a promising avenue for selectively reducing glucosinolate concentrations in seeds while preserving glucosinolate biosynthesis elsewhere. Targeting *GTRs*, particularly through downregulation using genome editing or RNA interference (RNAi) techniques, holds potential for enhancing the nutritional quality and stress resilience of *Brassica* crops. Notably, glucosinolate levels have been observed to rise under stress conditions, indicating a probable involvement of transporters in mediating this response. However, the specific role of glucosinolate transporters in regulating glucosinolate concentrations under stress and the major *GTRs* involved in their transportation remain inadequately understood.

In Arabidopsis, *GTR1* and *GTR2* have been identified as pivotal transporters responsible for loading glucosinolates into seeds. However, the evolutionary trajectory of *Brassica* crops, characterized by whole-genome triplication and subsequent gene duplications, has led to the emergence of multiple homologues of these genes. This genetic complexity poses challenges for manipulating glucosinolate transport in *Brassica* species [40,41,42,43]. The primary objective of this study was to identify key *GTR* genes in modulating stress-induced alterations in glucosinolate levels in *Brassica* crops. By elucidating the mechanisms through which these transporters influence glucosinolate accumulation under stress conditions, valuable insights can be gained for devising strategies aimed at augmenting glucosinolate content in *Brassica* seeds. Ultimately, such endeavors hold promise for the development of more nutritious and stress-tolerant varieties of *Brassica* crops.

This study undertook a comprehensive examination of *GTR* genes in *Brassica* species, employing a multi-faceted approach. Beginning with the retrieval of *GTR* gene and protein sequences utilizing Arabidopsis GTR1 and GTR2 templates, the investigation progressed through bioinformatics analyses, including phylogenetic, domain, motif, and promoter analyses. These analyses collectively confirmed the presence of transporter domains and regulatory elements associated with stress and metabolic regulation, implicating the involvement of *GTRs* in glucosinolate transportation. Subsequently, two *Brassica* species with divergent glucosinolate levels, *B. juncea* and *B. napus*, were subjected to NaCl stress, accompanied by biochemical profiling and quantitative real-time PCR to monitor *GTR* expression across various plant tissues. Statistical modelling techniques were then applied to discern the significant contributions of *GTRs* to glucosinolate concentrations with and without salt stress. This integrative approach yielded valuable insights into the evolutionary dynamics, structural characteristics, and functional roles of *GTR* genes in *Brassica* under stress conditions, offering implications for potential advancements in crop improvement strategies.

## 2. Materials and Methods

### 2.1. Plant Material and Growth Conditions

Two *Brassica* species with contrasting glucosinolate contents—*B. juncea* cv Super Raya and *B. napus* cv Westar—were used in this study. *B. juncea*, commonly known as Indian mustard or brown mustard, is a widely cultivated *Brassica* species in the Indian subcontinent as well as China [44]. It is better adapted to harsh climatic conditions, shows less pod shattering, and possesses a higher level of resistance against blackleg disease. It contains much higher levels of glucosinolates (up to 160 μmol per gram of fresh weight) compared with *B. napus* [45]. *B. napus* contains much lower levels of glucosinolates (<30 μmols of glucosinolates per gram of air-dried oil-free solid) and is a major oilseed crop of the Northern Hemisphere [42]. Seeds of these genotypes were obtained from the Oilseed Research Institute, Faisalabad, and sown in pots and kept under optimum growth conditions of 25/16 ± 2 °C day/night temperature, 16 h light/8 h dark period in the glass house. After successful germination, the plants were thinned to three plants/pot at uniform growth stage and were irrigated with 0.5× Hoagland solution containing 0 mM, 100 mM, or 200 mM NaCl. Plants were exposed to NaCl stress at the 2–3 leaf stage. Salt was applied in increments of 50 mM until the required concentration was achieved. The experiment was performed in a randomized complete block design (RCBD), with one replicate of each treatment per block. The experiment was repeated three times. Samples from different tissues, viz., roots, stems, leaves, open flowers, and siliques, were collected from the control and stressed plants for qPCR analysis. Only leaf samples were collected for biochemical and antioxidant assays. The samples were collected in liquid nitrogen and immediately stored at −80 °C until proceeding for RNA extraction and biochemical/antioxidant assays.

### 2.2. Bioinformatics Analyses

Protein sequences of Arabidopsis GTRs (GTR1 and GTR2) were retrieved from the TAIR database (arabidopsis.org, accessed on 14 May 2022). The AtGTR protein sequences were then used as query to retrieve the protein sequences from six *Brassica* species genomes, viz., *B. napus* (Dar V10.pep), *B. juncea* (tum V 2.0 pep), *B. carinata* (zd1 V 1.0 pep), *B. oleracea* (HDEM V 1.0 pep), *B. rapa* (Chiifu V 3.5 pep), and *B. nigra* (Ni100 V2.pep), available at the BRAD database (brassicadb.cn, accessed on 14 May 2022) using BLASTP analysis. The sequences having more than 90% query coverage, e-value of less than 1 × 10^−10^, and similarity of more than 80% were selected for further bioinformatics analyses. 

The locations of respective genes on chromosomes, gene length, transcript length, number of introns/exons, and protein length were obtained from the BRAD database. The ExPASy (SIB Swiss Institute of Bioinformatics|Expasy, https://www.expasy.org/, accessed on 20 May 2022) online resource was used to calculate different properties of the proteins including molecular weight (Da), isoelectric point (pI), and grand average of hydropathy (GRAVY) values. The subcellular localization of proteins was predicted using the WoLF PSORT (Protein Subcellular Localization Prediction (hgc.jp, accessed on 21 May 2022)) website. The phylogenetic tree was constructed using the unrooted neighbor-joining (NJ) method with 1000 bootstrap value employing MEGA7 (Home (megasoftware.net, accessed on 11 September 2022)) software and visualized using iTOL tree of life online tool (iTOL: Interactive Tree Of Life (embl.de, accessed on 11 September 2022)). 

For further bioinformatics analyses, only Arabidopsis, *B. juncea*, and *B. napus* protein and gene sequences were used. Protein sequence alignment and calculation of similarity percentage were carried out using MEGA7. All pairwise distances between protein sequences were also calculated in MEGA7 to infer divergence percentages. Non-synonymous–synonymous (K_a_/K_s_) ratios were calculated for the gene pairs evolving from the same node using Tbtools (TBtools bio.tools, https://bio.tools/tbtools, accessed on 17 August 2023) to find out the type of selection pressure experienced by the genes in the course of evolution. For gene structures, genomic and coding DNA sequences of Arabidopsis, *B. juncea*, and *B. napus* were retrieved from their respective genomes available at the BRAD database and submitted to the online gene structure display server (GSDS) (gao-lab.org, accessed on 19 September 2023) tool. Domain analysis was performed using NCBI CDD (conserved domain database) (NCBI conserved domain search (nih.gov, accessed on 17 October 2023)) to make sure each of the selected protein sequences possessed the conserved domains. Motif analysis was performed by submitting the protein sequences to the online MEME suite 5.5.4 (Introduction—MEME Suite (meme-suite.org, accessed on 20 October 2023)) tool based on “zero or one occurrence per sequence (zoops)” in classic mode for the identification of statistically significant motifs in the respective proteins. The motif length was set to a minimum of 15 and a maximum of 60 amino acids. The identified motif sequences were submitted to InterProScan (InterProScan—InterPro (ebi.ac.uk, accessed on 22 October 2023)) online tool to obtain their functional description.

For promoter region analysis and identification of cis-regulatory elements, a 2 kb upstream region of the respective genes was downloaded from the BRAD database. Cis-regulatory elements were identified using PlantCARE (a database of plant promoters and their cis-acting regulatory elements (ugent.be, accessed on 25 October 2023)) and visualized using Tbtools. 

### 2.3. Biochemical Analyses

Leaf samples were taken from the control and treated plants after the completion of salt stress treatment, for biochemical analysis. 

#### 2.3.1. Malondialdehyde (MDA) Content

MDA content was measured using the protocol described by [46]. Leaf tissue (0.5 g) was ground in 5 mL volume of 0.1% TCA solution followed by centrifugation at 12,000× *g* for 10 min to obtain supernatant. After that, 1 mL of supernatant was mixed with 4 mL of 0.5% TBA in 20% TCA solution and the reaction was incubated at 95 °C for 30 min. The reaction was stopped by placing the sample on ice. The absorbance was measured at 532 nm and 600 nm. Final MDA content was calculated using extinction coefficient 155 mM^−1^cm^−1^.

#### 2.3.2. Hydrogen Peroxide (H_2_O_2_) Content

A protocol published by [47] was used to determine H_2_O_2_ content. The leaf sample (1 g) was ground in 10 mL of 0.1% TCA solution followed by centrifugation at 12,000× *g* for 10 min. The reaction was prepared by adding 0.5 mL of each of the supernatant and potassium phosphate buffer and 1 mL of potassium iodide. Absorbance was measured at 390 nm and H_2_O_2_ was calculated by comparing the absorbance with the standard curve drawn from H_2_O_2_.

#### 2.3.3. Total Free Amino Acids (TFA) Content

The protocol devised by [48] was used for the estimation of TFAs. Leaf tissue (0.5 g) was ground in 5 mL of 0.1 M phosphate buffer (pH 7) and centrifuged at 12,000× *g* for 10 min. The reaction was prepared by adding 1 mL of each supernatant, 2% ninhydrin solution, and 10% pyridine and incubated at 100 °C for 30 min. The reaction was terminated by placing it on ice immediately and absorbance was measured at 570 nm. The TFA content was calculated using the leucine standard curve. 

#### 2.3.4. Total Soluble Protein (TSP) Content 

Total soluble proteins (TSPs) were determined by using a method developed by [49]. The leaf sample (0.5 g) was homogenized in 0.1 M phosphate buffer (pH 7) followed by centrifugation at 12,000× *g* for 10 min. Then, 2 mL of Bradford reagent was mixed with 200 µL of supernatant and the reaction was incubated at room temperature for 5 min. The absorbance was measured at 595 nm within 30 min. The TSP content was calculated by comparing the absorbance values with the standard curve drawn from BSA. 

#### 2.3.5. Phenolic Compounds 

Total phenolic content was measured using the method of [50]. Around 0.5 g leaf sample was extracted in 80% acetone followed by centrifugation at 12,000× *g* for 10 min. The reaction mixture was prepared by adding 1 mL of supernatant to 5 mL of FC reagent (1:10) and mixing well. Afterwards, 4 mL of Na_2_CO_3_ (7.5% *w*/*v*) was added and the reaction mixture was incubated at room temperature for 30 min. The absorbance was measured at 750 nm. Total phenolic content was calculated by comparing the absorbance values with the standard curve drawn from gallic acid. 

### 2.4. Antioxidant Assays

For antioxidant assays, 0.5 g leaf tissue was ground in 0.1 M phosphate buffer (pH 7.8) followed by centrifugation at 12,000× *g* and 4 °C for 10 min to obtain supernatant. The supernatant was then used for all antioxidant assays.

The NBT method was used for the determination of superoxide dismutase (SOD) content following the protocol of [51]. The 3 mL assay volume containing 50 mM phosphate buffer (pH 7.8), 2 mM EDTA, 9.9 mM methionine, 55 µM NBT, and 0.025% triton-X100 was prepared. Afterwards, 50–100 µL of enzyme extract and 20 µL of 1 mM riboflavin were added and the reaction was initiated by placing the test tubes under 15 W fluorescent light for 15 min. The absorbance was measured at 560 nm wavelength for 2 min. In the calculations, 50% inhibition in the photo-reduction of NBT was considered as 1 unit of SOD.

For peroxidase (POD) estimation, a protocol devised by [52] was followed. A 3 mL reaction was prepared containing 50 mM phosphate buffer (pH 5), 20 mM guaiacol, 40 mM H_2_O_2_, and 50–100 µL crude enzyme extract. The absorbance was measured at 470 nm for 2 min. An extinction coefficient of 26.6 mM^−1^cm^−1^ was used to calculate the enzyme activity.

Catalase (CAT) assay was performed according to [53]. The reaction volume of 3 mL was prepared containing 50 mM phosphate buffer (pH 7), 5.9 mM H_2_O_2_, and 50–100 µL crude enzyme extract. The optical densities were measured at 240 nm for 2 min and enzyme activity was calculated using extinction coefficient 0.036 mM^−1^cm^−1^.

For ascorbate peroxidase (APX) activity, a protocol developed by [54] was followed. The reaction volume (3 mL) was prepared containing 50 mM phosphate buffer (pH 7), 0.1 mM EDTA, 12 mM H_2_O_2_, 0.25 mM ascorbic acid, and 50–100 µL enzyme extract. The absorbance was measured at 290 nm for 2 min. Enzyme activity was measured using an extinction coefficient of 2.8 mM^−1^cm^−1^.

### 2.5. RNA Extraction, cDNA Synthesis and qPCR Analysis

RNA extraction of plant tissues was performed manually using a tri-reagent from Molecular Research Center lnc. (MRC) as per supplier instructions. The quality and integrity of RNA were confirmed using a NanoDrop™ 2000/2000c spectrophotometer (Thermo Scientific Inc., Waltham, MA, USA) and 1.2% agarose gel electrophoresis. Residual DNA contamination was removed by treating RNA with RNase-free DNase I (Thermo Scientific Inc.) and dilutions were made so that each sample contained an equal amount of RNA. For this process, 5 µg of RNA was used to synthesize cDNA using a RevertAid First Strand cDNA Synthesis Kit (Thermo Scientific Inc.). Four genes from *B. juncea* were selected based on their higher CPKM (counts per kb per million reads) values as reported by [42]. The genes from *B. napus* were selected based on the highest protein sequence similarity with the selected *B. juncea* genes. Gene-specific primers were designed using the Primer 3 online tool and synthesized commercially (Table S1). A Bio-Rad CFX96 touch real-time PCR detection system was used for performing qPCR. The qPCR reaction was prepared by adding 6.25 µL of 2X bright green master mix from Applied Biological Material lnc., Richmond, BC, Canada, 0.25 µL of each of the forward and reverse primer stock (10 µM), 3–4 µL of cDNA template, and nuclease-free water to make a total volume of 12.5 µL. PCR conditions followed were 95 °C for 5 min followed by 40 cycles of 95 °C for 30 s, 55 °C for 30 s, and 72 °C for 30 s. Melt curve analysis was then performed by increasing temperature from 55 °C to 95 °C and finally keeping at 95 °C for 5 s. Expression of target genes was normalized relative to expression of the *Actin* gene as an internal control. The experiment was performed in triplicate. Relative expression of the target genes was calculated by the 2^−∆∆CT^ method using the following formulas:

∆CT(test) = CT(target, test) − CT(reference, test)

∆CT(calibrator) = CT(target, calibrator) − CT(reference, calibrator)

∆∆CT = ∆CT(test) − ∆CT(calibrator)

2^−∆∆CT^ = Normalized expression ratio

### 2.6. Measurement of Seed Glucosinolates Content

Seed glucosinolates content was measured using the non-destructive near-infrared reflectance spectroscopy (NIRS) method. For this purpose, the DA 7250 At-Line NIR process analyzer was used. The instrument was calibrated using an oilseed of known glucosinolate concentrations. As a statistical approach. partial least square regression (PLSR) was used to establish the relationship between spectral data and glucosinolate concentration. Approx. 30 g seed was used for the measurement of glucosinolate contents. 

### 2.7. Statistical Analyses

Statistical analyses were performed using R Environment 4.1.3 for Windows. The metabolite, glucosinolate, and gene expression data were analyzed for significance using one- or two-way ANOVA followed by Tukey’s HSD (honest significant difference) post hoc test for pairwise comparison of means between different groups. Generalized linear regression models were developed using a forward selection method to determine the key genes involved in loading glucosinolates into the seed. Starting with a null model, forward selection identified the most significant genes in each plant part to include in the model, based on the Akaike Information Criterion (AIC) value. The process was repeated multiple times for each gene, selecting the most effective models with n + 1 genes compared with the current model with n genes, based on AIC value. The process was stopped when none of the remaining genes significantly improved the estimation of glucosinolate concentration at a 5% significance level [55].

## 3. Results

### 3.1. Bioinformatics Analysis

#### 3.1.1. Identification of GTR Genes in *Brassica* Complex

The putative protein sequences corresponding to *GTR* genes were sourced from *Brassica* species using Arabidopsis sequences of GTR1 and GTR2 proteins as templates. A total of 54 putative protein sequences were identified, with 27 associated with each template. In the case of GTR proteins from Arabidopsis, six and twelve sequences were retrieved from each diploid and amphidiploid species, respectively. The characteristics of these proteins, including amino acid length, protein weight, isoelectric point, and GRAVY values, exhibited ranges of 526 to 740 amino acids, 57.9 to 81.4 kilodaltons, 8.61 to 9.54, and 0.106 to 0.452, respectively. Notably, all GTR proteins were consistently observed to be localized on the plasma membrane (Table S2).

#### 3.1.2. Phylogenetic Analysis of GTR Proteins in *Brassica* Complex

**Figure 1:**
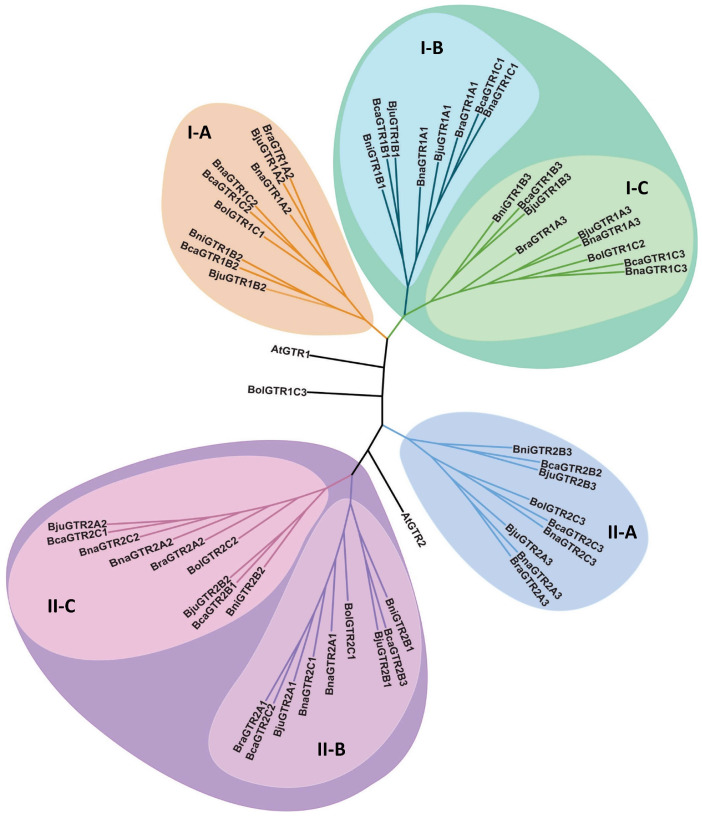
Phylogenetic tree of GTR proteins from six *Brassica* species of U’s triangle (*B. carinata*, *B. juncea*, *B. napus*, *B. nigra*, *B. oleracea*, and *B. rapa*) and Arabidopsis. A phylogenetic tree was constructed in MEGA7 using the unrooted neighbor-joining (NJ) method and 1000 bootstrap values. Different phylogenetic clades are represented in different colours. The numbers I and II represent GTR1 and GTR2 protein groups, respectively, and A, B, and C represent further phylogenetic subclades among GTR1 and GTR2 proteins. The accession numbers for query proteins are given as follows: AtGTR1 (AT3G47960.1), BolGTR1C1 (BolC3t18961H), BolGTR1C2 (BolC1t03184H), BolGTR1C3 (BolC8t51846H), BniGTR1B1 (BniB08g061180.2N), BniGTR1B2 (BniB06g024230.2N), BniGTR1Bn3 (BniB05g052360.2N), BraGTR1A1 (BraA06g019540.3.5C), BraGTR1A2 (BraA06g025210.3.5C), BraGTR1A3 (BraA01g025200.3.5C), BnaGTR1A1 (A06p19700.1_BnaDAR), BnaGTR1A2 (A06p27390.1_BnaDAR), BnaGTR1A3 (A01p25730.1_BnaDAR), BnaGTR1C1 (C03p72210.1_BnaDAR), BnaGTR1C2 (C03p66900.1_BnaDAR), BnaGTR1C3 (C01p32480.1_BnaDAR), BjuGTR1A1 (BjuVA06G19500), BjuGTR1A2 (BjuVA06G26150), BjuGTR1A3 (BjuVA01G26580), BjuGTR1B1 (BjuVB08G47510), BjuGTR1B2 (BjuVB06G23560), BjuGTR1B3 (BjuVB05G42900), BcaGTR1B1 (BcaB01g00859), BcaGTR1B2 (BcaB02g10681), BcaGTR1B3 (BcaB05g23096), BcaGTR1C1 (BcaC01g01757), BcaGTR1C2 (BcaC01g02298), BcaGTR1C3 (BcaC09g49832), AtGTR2 (AT5G62680.1), BolGTR2C1 (BolC2t12292H), BolGTR2C2 (BolC3t18666H), BolGTR2C3 (BolC9t53791H), BniGTR2B1 (BniB04g002130.2N), BniGTR2B2 (BniB06g010940.2N), BniGTR2B3 (BniB07g041760.2N), BraGTR2A1 (BraA02g045700.3.5C), BraGTR2A2 (BraA06g027380.3.5C), BraGTR2A3 (BraA09g007530.3.5C), BnaGTR2A1 (A02p42460.1_BnaDAR), BnaGTR2A2 (A06p29420.1_BnaDAR), BnaGTR2A3 (A09p07810.1_BnaDAR), BnaGTR2C1 (C02p62380.1_BnaDAR), BnaGTR2C2 (C03p64110.1_BnaDAR), BnaGTR2C3 (C09p08780.1_BnaDAR), BjuGTR2A1 (BjuVA02G47610), BjuGTR2A2 (BjuVA06G28410), BjuGTR2A3 (BjuVA09G07610), BjuGTR2B1 (BjuVB04G01960), BjuGTR2B2 (BjuVB06G11810), BjuGTR2B3 (BjuVB07G18470), BcaGTR2B1 (BcaB02g09673), BcaGTR2B2 (BcaNung01385), BcaGTR2B3 (BcaB07g29850), BcaGTR2C1 (BcaC01g02504), BcaGTR2C2 (BcaC03g18168), BcaGTR2C3 (BcaC04g23397).

The phylogenetic tree constructed for GTR proteins across six *Brassica* species and Arabidopsis (Figure 1) reveals distinct clustering patterns, forming two major clades, Clade 1 and Clade II, delineated by GTR1 and GTR2, respectively, indicating a divergent evolution of these orthologues in *Brassica*. Within each of these primary groups, further subdivisions occur, resulting in the formation of three distinct subgroups. Remarkably, each subgroup within both clades encompasses a cohesive set of GTR orthologues. Within these groups, an intriguing pattern emerges where GTR orthologues of one type appear together. Based on this grouping, three distinct variants were identified within each clade, each exclusively present in subgroups labelled A, B, and C, respectively. This organization underscores the evolutionary relationships and divergence of GTR proteins among the studied *Brassica* species and Arabidopsis, suggesting both conserved and divergent functions across these taxa. Such a detailed categorization sheds light on the intricate evolutionary dynamics and functional diversification of *GTR* genes within this plant lineage. 

In addition to the observed clustering patterns, another notable trend emerged regarding the relationship between tetraploid and diploid varieties. Interestingly, the GTRs of tetraploid species exhibit a higher degree of relatedness to each other compared with their diploid parental counterparts. This observation suggests a possible divergence in GTR evolution following polyploidization events, wherein tetraploid species may have undergone distinct evolutionary trajectories leading to greater similarity amongst their GTRs. This phenomenon underscores the complex interplay between polyploidization events and the evolutionary dynamics of gene families within *Brassica* species and Arabidopsis. Further investigation into the functional implications of these divergent evolutionary paths could provide valuable insights into the adaptive mechanisms underlying the evolution of GTRs in polyploid plant lineages.

A multiple sequence alignment of GTR proteins from Arabidopsis, *B. juncea*, and *B. napus* was conducted using the MUSCLE online alignment tool to explore the similarities and differences at the amino acid level (Figure S1). The sequence similarity (%) between the proteins ranged from 73.5% to 99.8%, indicating high similarities among the GTRs (Table 1). To illustrate the distinctions among the GTRs, divergence percentages were calculated through pairwise distances. Remarkably similar proteins exhibited low divergence percentages, while those with greater differences showed higher values. Additionally, the non-synonymous–synonymous (K_a_/K_s_) substitution ratio was calculated to investigate the selection pressure on *GTR* genes. All gene pairs showed a K_a_/K_s_ ratio of less than 1, suggesting the prevalence of purifying selection, which indicates the removal of deleterious alleles during evolution (Table S3).

**Figure 2:**
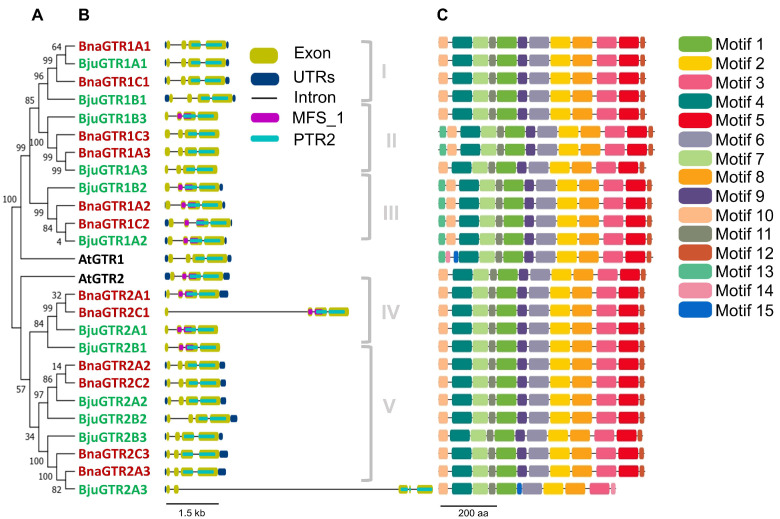
Phylogenetic relationship, intron/exon organization, domain, and motif analysis of GTR proteins from *B. juncea*, *B. napus*, and Arabidopsis. (**A**) A phylogenetic tree of GTR proteins was constructed in MEGA7 using full-length protein sequences of *B. juncea*, *B. napus*, and Arabidopsis, employing the unrooted neighbor-joining (NJ) method and 1000 bootstrap values. Black, red, and green colors represent Arabidopsis, *B. juncea*, and *B. napus* proteins, respectively. (**B**) domain analysis and intron/exon organization of GTR proteins from *B. juncea*, *B. napus*, and Arabidopsis. Domain analysis was performed by submitting the full-length protein sequences to NCBI- Conserved Domain Database (NCBI-CDD), further selecting the Pfam v34.0—19178 PSSMs database. Intron/exon organization of *GTR* genes’ and domains’ positions were drawn in Gene Structure Display Server (GSDS) by submitting the full length and coding DNA sequences of *GTR* genes and the BED file for the location of domains, selecting the proteins’ ordinates. Dark blue-colored rounded rectangles represent untranslated regions (UTRs), and solid black lines and yellow-colored rounded rectangles represent introns and exons, respectively. Green- and purple-colored rounded rectangles represent PTR2 and MFS_2 domains, respectively. Numbers I to V indicate different phylogenetic groups. (**C**) Motif analysis was performed using MEME suite 5.5.4 and a total of 15 statistically significant motifs were identified among the query proteins, which are represented by different colors as given in the legend. The accession numbers of the query proteins are given as follows: AtGTR1 (AT3G47960.1), BnaGTR1A1 (A06p19700.1_BnaDAR), BnaGTR1A2 (A06p27390.1_BnaDAR), BnaGTR1A3 (A01p25730.1_BnaDAR), BnaGTR1C1 (C03p72210.1_BnaDAR), BnaGTR1C2 (C03p66900.1_BnaDAR), BnaGTR1C3 (C01p32480.1_BnaDAR), BjuGTR1A1 (BjuVA06G19500), BjuGTR1A2 (BjuVA06G26150), BjuGTR1A3 (BjuVA01G26580), BjuGTR1B1 (BjuVB08G47510), BjuGTR1B2 (BjuVB06G23560), BjuGTR1B3 (BjuVB05G42900), BnaGTR2A1 (A02p42460.1_BnaDAR), BnaGTR2A2 (A06p29420.1_BnaDAR), BnaGTR2A3 (A09p07810.1_BnaDAR), BnaGTR2C1 (C02p62380.1_BnaDAR), BnaGTR2C2 (C03p64110.1_BnaDAR), BnaGTR2C3 (C09p08780.1_BnaDAR), BjuGTR2A1 (BjuVA02G47610), BjuGTR2A2 (BjuVA06G28410), BjuGTR2A3 (BjuVA09G07610), BjuGTR2B1 (BjuVB04G01960), BjuGTR2B2 (BjuVB06G11810), BjuGTR2B3 (BjuVB07G18470).

The phylogenetic tree was also developed with the same proteins in MEGA7 using the NJ method (Figure 2A) to explain the inter-relationship of Arabidopsis, *B. juncea*, and *B. napus* only and to relate their phylogenetic relationship with their gene structure, domain analysis, and motif analysis.

#### 3.1.3. Intron/Exon Composition of GTR Genes

The gene structures of *GTR* genes were determined using the GSDS online tool, revealing a conserved pattern of exon numbers across all genes, mirroring the four-exon structure seen in Arabidopsis *GTR* genes (Figure 2B). Notably, *BjuGTR2A3* displayed a unique pattern with an additional, comparatively small exon in addition to the four parental exons. However, variations in gene sizes were observed, particularly in genes with long introns such as *BnaGTR2C1*, *BjuGTR2B2*, and *BjuGTR2A3*. Interestingly, while Arabidopsis *GTRs* contained untranslated regions (UTRs), not all *Brassica* genes exhibited this feature. Furthermore, genes within the same phylogenetic groups generally shared similar genetic organizations, indicative of their close evolutionary relationships. Exceptions were observed, notably in Group I where all genes contained UTRs, contrasting with the absence of UTRs in all genes from Group II. Similarly, while most genes in Groups III and V included UTRs, exceptions like *BjuGTR1B2* in Group III lacked a 5’ UTR, and *BjuGTR2A3* lacked a 3’ UTR, highlighting additional structural diversity within these phylogenetic clusters.

#### 3.1.4. Domain Analysis of GTR Proteins

Protein sequences underwent domain analysis using the NCBI Conserved Domain Database (NCBI-CDD) and the Pfam v340-19178 PSSMs database. All GTR proteins were found to feature the conserved peptide transporter (PTR2, pfam00854) domain, essential for transport activity. Additionally, some proteins contained the major facilitator superfamily (MFS_1, pfam07690) domain, implicated in transport functions. In Arabidopsis, MFS_1 was exclusive to GTR2, while in *B. juncea* and *B. napus*, certain GTR proteins possessed this domain. The clustering of proteins with similar gene structures and domain presence in phylogenetic groups suggests close relationships. Both the PTR2 and MFS_1 domains belong to the major facilitator superfamily (MFS superfamily, cl28910), facilitating diverse molecule transport across membranes (Figure 2B).

#### 3.1.5. Motif Analysis of GTR Proteins

Motif analysis was conducted using MEME suit v5.5.4 and visualized with Tbtools. Conserved motifs were identified in all GTR proteins, with some exceptions (Figure 2C). Motifs 1-4, 6–8, and 11 were conserved across all GTR proteins. Motif lengths varied, with motifs 1–4, 6, and 8 being the longest at 60 amino acids (aas) while motifs 14 and 15 were the shortest at 15 aas. Some motifs were absent in specific proteins, such as motif 10 in AtGTR1 and motifs 5, 9, and 12 in BjuGTR2A3. Additionally, unique motifs were found in AtGTR1 and BjuGTR2A3.

For functional annotation, motif sequences were analyzed using InterProScan. Motifs 1-3 and 8 were classified as members of the proton-dependent oligopeptide transporter family (POT, IPR000109), also known as the peptide transporter family (PTR2, pfam00854), and are homologous to the MFS general substrate transporter-like domain (IPR036259). Motifs 4, 5, and 7 showed homology to the MFS general substrate transporter-like domain but were not predicted by InterProScan. Motifs 3 and 7 also exhibited features of the transmembrane helix (TMHMM: TMhelix), important for receptor function across lipid bilayers. These motifs collectively contribute to transmembrane transporter activity. Motifs 10 and 13 were not classified into specific families but contained the MOBIDB_LITE feature, associated with intrinsically disordered regions involved in cell signalling. Motifs 6, 9, 11, 12, 14, and 15 were unique with unknown functions, based on InterProScan results.

#### 3.1.6. Cis-Regulatory Elements Analysis of GTR Genes

**Figure 3:**
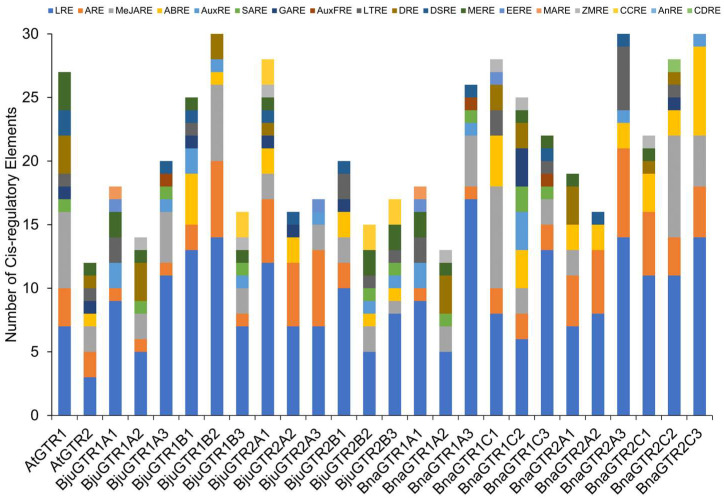
Cis-acting elements analysis of the promoter regions of the GTR genes from Arabidopsis, *B. juncea*, and *B. napus*. The cis-regulatory element analysis was performed by submitting the 2 kb upstream regions of the GTR genes to the PlantCARE database. Different cis-acting elements are represented by different colored rectangles, as per legend. The cis-acting elements are abbreviated as follows: auxin-related responsive element (AuxRE), light-responsive element (LRE), low-temperature related element (LTRE), endosperm expression regulatory element (EERE), meristem specific activation regulatory element (MARE), anaerobic induction element (ARE), meristem specific regulatory element (MERE), zein metabolism regulatory element (ZMRE), drought inducibility element (DRE), abscisic acid-responsive element (ABRE), methyl jasmonate-responsive element (MeJARE), gibberellic acid-responsive element (GARE), defense- and stress-related element (DSRE), circadian control regulatory element (CCRE), salicylic acid-responsive element (SARE), responsive element for auxin free medium (AuxFRE), cell division regulatory element (CDRE), anoxic specific inducibility element (AnRE).

Promoter region analysis using the PlantCARE database revealed multiple light-responsive elements (LRE) in all *GTR* genes, with a maximum of 14 copies in some genes (Figure 3). The presence of anaerobic responsive elements (ARE) was widespread, except in a few genes. Hormone-related cis-regulatory elements like abscisic acid-responsive element (ABRE) and methyl jasmonate-responsive element (MeJARE) were abundant, while others like salicylic acid-responsive element (SARE) were less frequent. Stress-related elements showed uneven distribution, with the low-temperature regulatory element (LTRE) being the most common. Tissue-specific expression elements such as meristem expression-related element (MERE) were present in most promoters, indicating tissue-specific regulation. Other regulatory elements such as zein metabolism-related elements (ZMRE) and circadian clock-related elements (CCRE) were also identified, highlighting the diverse roles of GTR genes in plant metabolism, growth, development, and stress responses (Figure 3).

### 3.2. Effect of NaCl Stress on Different Metabolites

**Figure 4:**
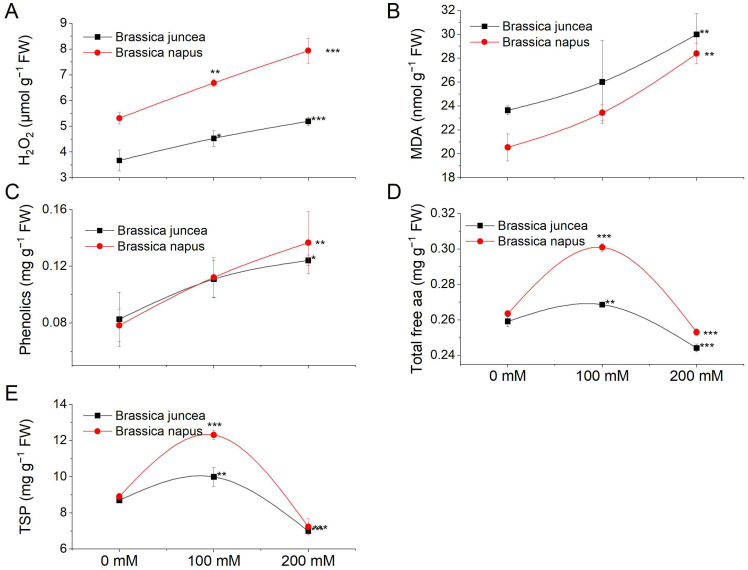
Effect of NaCl stress on the biochemical parameters of *B. juncea* and *B. napus*. *B. juncea* (black lines) and *B. napus* (red lines) plants were grown under 0, 100, and 200 mM NaCl stress and were used for sample collection. (**A**) H_2_O_2_ content, (**B**) MDA content, (**C**) phenolic compounds, (**D**) total free amino acids (TFAs), (**E**) total soluble proteins (TSPs). Data points represent the means ± SD of three biological replicates. Significant differences are shown with different asterisks (***, **, * at *p* = 0.0001, 0.001, and 0.01, respectively.).

We evaluated various metabolites, including hydrogen peroxide (H_2_O_2_), malondialdehyde (MDA), total phenolics, total free amino acids (TFA), and total soluble proteins (TSP), to understand oxidative stress, antioxidant capacity, and cellular responses (Figure 4). H_2_O_2_ concentration, indicating oxidative stress, increased significantly (*p* < 0.05) under salt stress in both species, with *B. napus* showing a more pronounced increase (Figure 4A). Lipid peroxidation, measured via MDA, increased significantly (*p* = 0.0001) under salt stress, particularly in *B. napus* (Figure 4B). Total phenolics increased significantly at higher NaCl concentrations (Figure 4C). TFA concentration rose dramatically at 100 mM salt concentration, decreasing at 200 mM (Figure 4D). TSP levels increased at 100 mM stress but decreased at 200 mM in both species (Figure 4E). The species, NaCl, and their interaction effects on these metabolites are detailed in Table S4a.

### 3.3. Effect of NaCl on Enzymatic Antioxidant Activities

**Figure 5:**
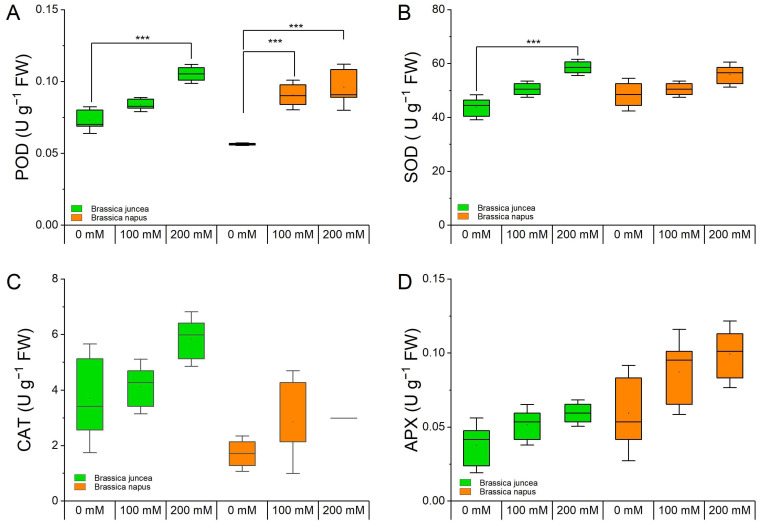
Effect of NaCl stress on the enzymatic antioxidant activities in *B. juncea* and *B. napus*. *B. juncea* (green box) and *B. napus* (orange box) plants were grown under 0, 100, and 200 mM NaCl stress and were used for sample collection. (**A**) Peroxidase, (**B**) superoxide dismutase, (**C**) catalase, (**D**) ascorbate peroxidase. Data points represent the means ± SD of three biological replicates (Tukey’s HSD, *p* < 0.05). Significant differences are shown with different asterisks (***, at *p* = 0.0001).

The activities of different antioxidant enzymes such as peroxidase (POD), superoxide dismutase (SOD), catalase (CAT), and ascorbate peroxidase (APX) in *B. juncea* and *B. napus* plants under varying NaCl concentrations (0, 100, and 200 mM) were measured to assess the plants’ responses to cope with the salinity stress (Figure 5).

The POD activity was significantly increased (*p* ≤ 0.0001) under salt stress conditions in both species. The increase in POD levels was more pronounced in *B. juncea* (23.5% and 54.4% at 100 and 200 mM, respectively) compared with that in *B. napus* (17.6% and 28% at 100 and 200 mM, respectively) (Figure 5A). The SOD activity was also increased in both species in a dose-dependent manner. The changes in *B. juncea* were much higher than those in *B. napus* (Figure 5B). Changes in CAT and APX activities were also observed but they were statistically non-significant (Figure 5C,D). The effects of species, NaCl treatment, and their interaction are indicated in Table S4b.

### 3.4. Effect of NaCl on Selected GTR Genes in Different Tissues

**Figure 6:**
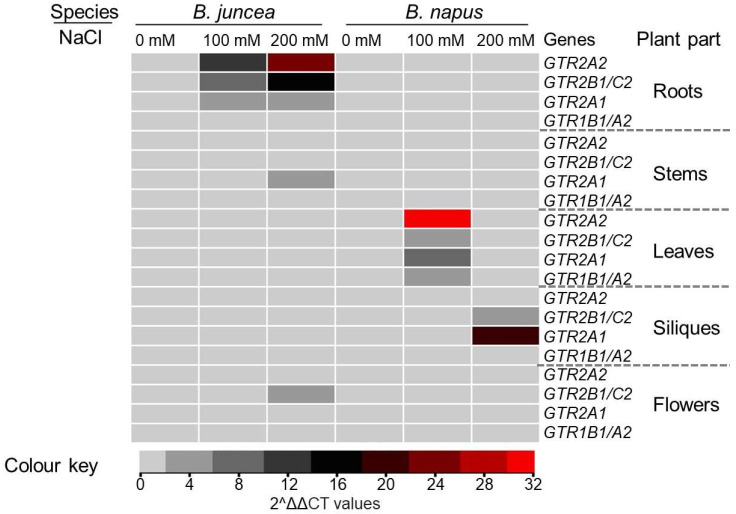
The expression of *GTR* genes in different tissues of *B. juncea* and *B. napus* under NaCl stress. Each data point shows the mean values obtained from three biological replicates.

Salinity stress is a major abiotic stress affecting *Brassica* yield and quality. This experiment was designed to investigate the effects of salinity on the expression of *GTR* genes in different tissues involved in the transport of glucosinolates into seed. Previously, the expression of several *GTR* genes in different parts of the plant has been reported [42]. Based on values reported in the literature, 4 out of the 12 *GTR* homologues present in *Brassica* were selected to investigate their contribution to glucosinolate concentration under NaCl stress. The *GTR* genes displayed variable expression patterns under mild and high salinity levels and in all tissues under investigation. The expression of *GTR* genes varied under mild and high salinity levels in all tissues examined (Figure 6). In *B. juncea*, *BjGTR1B1* expression increased in roots but remained unaffected elsewhere. *BnGTR1A2* expression decreased in roots, stems, and flowers, but increased in siliques of *B. napus* at both salinity levels. *GTR2A2* exhibited similar patterns in siliques and flowers but opposite patterns elsewhere in both species. *GTR2A1* was highly induced in *B. juncea* roots under increased NaCl levels while showing mild changes in *B. napus*. *BjGTR2B1* expression increased in roots, stems, and flowers of *B. juncea*, but decreased in leaves and siliques. *BnGTR2C2* expression was significantly reduced in roots, flowers, and siliques of *B. napus* under NaCl stress.

### 3.5. Determining the Effect of GTR Gene Expression on the Loading of Glucosinolates into the Seed

Utilizing the forward stepwise selection method, statistical models were constructed to elucidate the influence of specific genes on seed glucosinolate (Table 2). These models shed light on the distinct roles of specific *GTR* genes in mediating glucosinolate transportation in *Brassica* species. Notably, *GTR2A2* demonstrated substantial expression in both stems (52.57%) and roots (60.52%), suggesting its crucial involvement in facilitating glucosinolate transport within these tissues. Despite *GTR2A2*’s expression not reaching statistical significance (*p* > 0.05), its inclusion in the model lowered the Akaike information criterion (AIC) value from 104.06 to 102.76. The Akaike information criterion (AIC) is a measure used for model selection that balances the goodness of fit of the model with its complexity. When comparing two models, a difference in AIC values (ΔAIC)~2 is generally considered substantial evidence in favor of the model with the lower AIC value. *GTR2B1/C2* exhibited significant expression exclusively in flowers (*p* = 0.001), indicating its role in regulating glucosinolate loading during reproductive phases. *GTR2A1* displayed notable expression in flowers (45.33%) and roots (58.82%), suggesting its dual influence on glucosinolate transport in both reproductive and underground tissues. Finally, *GTR1A2*/B1 showed significant expression in roots (*p* = 0.04), emphasizing its importance in glucosinolate loading within tissues responsible for anchorage and structural support. Despite *GTR1A2*/B1’s lack of statistical significance in the stem, its inclusion in the model led to a reduction in AIC value from 106.00 to 103.43, indicating its influence.

### 3.6. Predicting the Effect of NaCl Stress on Glucosinolates Content of B. napus and B. juncea

A two-factor analysis of variance (ANOVA) was conducted to investigate the significance of species and NaCl (salt) stress on seed glucosinolate concentration. Species variation reflects the genetic diversity within the *Brassica* genus, which can lead to differences in glucosinolate profiles among different species. On the other hand, NaCl stress represents an environmental factor that can trigger physiological responses in plants, potentially altering their biochemical composition, including glucosinolate levels.

The ANOVA revealed that both species and NaCl stress significantly (*p* < 0.05) affected seed glucosinolate concentration. Species accounted for the majority (96%) of the variance in glucosinolate content (*F* = 1900.88, *p* = 2 × 10^−16^; Table S4d), highlighting the strong influence of genetic factors on this trait. This observation reflects the importance of genotype in determining the chemical composition of *Brassica* seeds. Understanding the genetic basis of glucosinolate production could facilitate breeding programs aimed at developing cultivars with enhanced nutritional quality and stress tolerance. While species had a dominant effect, NaCl stress also had a significant but minor impact, explaining only 0.04% of the variance in seed glucosinolate concentration (*F* = 4.50; *p* = 0.014). This suggests that while environmental stressors like salt can influence glucosinolate levels to some extent, their contribution is relatively small compared with genetic factors. Nonetheless, understanding how environmental stressors interact with genetic factors to modulate glucosinolate production is essential for developing resilient crop varieties capable of thriving under adverse conditions.

**Figure 7:**
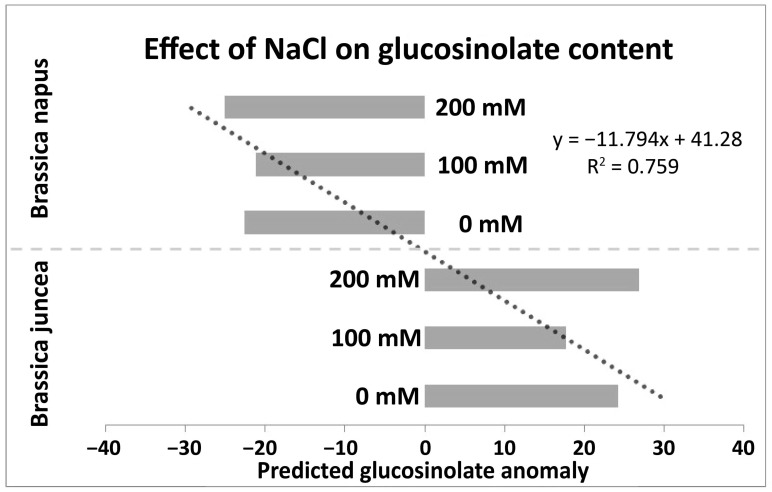
Predicting the effect of NaCl stress on glucosinolate content in *Brassica*. Bars represent the coefficient of the correlation for each stress level.

In order to delve deeper into the impact of NaCl on seed glucosinolates, we employed a generalized linear model. In this model, the species variable was intentionally omitted as a term. By excluding species as a variable, we aimed to isolate the specific influence of each NaCl stress level on seed glucosinolates. This approach allowed us to discern and quantify the individual effects of different NaCl concentrations on the composition and abundance of seed glucosinolates, providing insights into the underlying mechanisms of response to salinity stress. (Figure 7). The model revealed contrasting responses of each species to NaCl stress. In *B. juncea*, there was a positive correlation between NaCl stress levels and seed glucosinolate content, indicating that higher salt concentrations led to increased glucosinolate production. This response might be interpreted as a stress-induced defence mechanism, where the plant produces more glucosinolates to protect itself under adverse environmental conditions. Conversely, in *B. napus*, the opposite trend was observed, with seed glucosinolate content decreasing as NaCl concentrations increased. This unexpected result suggests a potentially different physiological response in *B. napus* compared with *B. juncea* when subjected to salt stress.

Overall, these findings highlight the complex interplay between genetic factors, environmental stressors, and biochemical pathways regulating glucosinolate production in *Brassica* plants. Understanding these dynamics is crucial for developing sustainable agricultural practices and breeding strategies to enhance the nutritional quality and stress resilience of *Brassica* crops.

## 4. Discussion

*Brassica* crops are known as an important source of nutritious vegetable oil and a protein-rich animal feed [56,57]. Yet, the existence of anti-nutrients such as erucic acid in seed oil and glucosinolates in the seed meal renders them unsuitable for human and animal consumption due to health concerns [58,59]. Glucosinolates are defence compounds and their synthesis takes place in different parts of the plant and are stored in the seed [60]. The transporters involved in the glucosinolate loading into the seed are under intensive research as they offer a means to selectively reduce glucosinolate content in the seed, without compromising their biosynthesis [39,42,43]. Reduction of glucosinolates is often a requirement for developing seed cakes for animal consumption [43,61]. In this study, we have conducted a genome-wide analysis of the key genes involved in the transport of glucosinolates in two distinct *Brassica* species that exhibit significant differences in their glucosinolate contents under NaCl stress. 

The two GTR proteins, GTR1 and GTR2, identified in Arabidopsis were used as queries to identify their orthologs in *B. juncea* and *B. napus*. The BLAST search yielded six orthologs in diploid species and twelve orthologs in amphidiploid *Brassicas*. The expansion of the *GTR* gene family can be attributed to the whole genome triplication that took place during the evolution of the *Brassicaceae* family, occurring approximately 9–15 million years ago [62,63]. This event preceded the divergence into diploid *Brassica* species, followed by additional polyploidization in the case of amphidiploid *Brassicas* [64,65]. The species-specific divergent evolution of *GTR* genes could possibly due to the duplication and polyploidization events occurring in their evolutionary path. Duplication and polyploidization are significant contributors to plant speciation and divergence as they result in the creation of novel gene functions, and neo-functionalization of duplicated genes as well as recombination [66]. Furthermore, transpositions and structural variation might also play a role in the divergent evolution of genes [67]. Genome-wide studies of various gene families in *Brassicaceae* have consistently indicated their expansion compared with Arabidopsis. Notable examples are not limited to but include *APX*, *NPR*, *DOF*, shattering genes, *SOD*, and *PEBP* [68,69,70,71,72,73]. Regarding amphidiploids, the presence of twelve genes suggests these species inherited six genes from each of their parental species, corresponding to the *GTR* genes found in Arabidopsis [42]. This is in line with the previous research conducted on other gene families of *B. juncea* which showed that the allotetraploid *Brassicas* attained genes approximately three times more than Arabidopsis [72]. Similar observations of gene duplications have been reported in other polyploid crops, such as cotton and wheat [74,75]. 

Phylogenetic, gene structure, and motif analyses of GTR proteins revealed that the majority of the *GTR* genes in *B. juncea* and *B. napus* retained their parental intron/exon organization as observed in Arabidopsis, with some exceptions (Figure 2). These findings were similar to those reported for the *BjJAZ* gene family in *B. juncea* [76]. A similar conservation of structural elements in the *GTR* gene family has been reported for other plant species as well, such as *Camelina sativa* [77]. Domain analysis of GTR protein sequences revealed a high degree of conservation for the crucial functional domain PTR2 in GTR proteins. However, some of the proteins also attained new domains such as MFS_1, further adding functional diversity to the GTR proteins [78,79]. Previously, these domains were found to be responsible for the broad glucosinolate specificity of *GTR* genes with no discrimination against amino acid side chains that are aliphatic or indolic, etc. [41]. However, a new *GTR3* gene has been identified in Arabidopsis which was found to be specifically involved in the transport of indolic glucosinolates [80]. The promoter analysis of *GTR* genes (Figure 3) indicated the presence of various cis-regulatory elements, which are implicated in the transcriptional regulation of gene expression across a range of biological processes. These processes include developmental processes, responses induced by hormones, and stress-related responses [81,82]. The presence of cis-regulatory elements associated with signalling molecules like JA, SA, and related elements suggests a role for *GTR* genes in stress conditions, highlighting a degree of specificity. This observation reinforces the concept of neofunctionalization occurring after genome duplication and triplication during speciation and evolution [72]. 

Biochemical analyses for the accumulation of various metabolites such as H_2_O_2_ and MDA revealed the sensitivity of genotypes to NaCl stress conditions. For example, the levels of MDA and H_2_O_2_ were more pronounced in *B. napus*, indicating the sensitivity of this species towards NaCl stress compared with *B. juncea*, which maintained relatively lower levels of both the stress parameters. In Arabidopsis, the increased H_2_O_2_ levels resulted in the induction of myrosinase-related genes which are involved in the hydrolysis of glucosinolates [83]. Cell membrane damage caused by the increased MDA content may also act as the signal for the activation of myrosinase enzymes to initiate glucosinolate breakdown [84,85]. Likewise, the accumulation of total phenolics—non-enzymatic antioxidants that help scavenge ROS—was also higher in *B. napus* compared with *B. juncea* plants exposed to stress. Regarding osmoregulatory compounds like total free amino acids and total soluble proteins, both *Brassica* species exhibited an increase in content at 100 mM NaCl, followed by a sharp decline as the stress intensified to 200 mM NaCl. This rise in solutes suggests the species’ adaptive response to survive under stress conditions by producing elevated levels of these solutes during mild NaCl stress, aiming to prevent osmotic imbalance induced by the stress [86]. However, the decrease in the content of these compounds at higher NaCl levels implies a shift in cellular processes toward the production of secondary metabolites, including glucosinolates (Figure 4) [87,88]. In *Brassica* species, these metabolites serve as crucial defence compounds, especially under salt stress, to protect cells from potential permanent damage. Reduction of glucosinolate contents in Arabidopsis through RNAi rendered plants highly sensitive to oxidative stress due to reduced levels of antioxidant proteins [89]. Perhaps that could be a reason for a stronger antioxidant repertoire of *B. juncea* plants to NaCl stress compared with *B. napus*. The *B. juncea* plants demonstrated elevated activities of antioxidant enzymes, including POD, SOD, and CAT, in response to NaCl stress, suggesting a robust defence against oxidative stress. This heightened antioxidant response could be attributed to the species’ higher accumulation of glucosinolates, known for their involvement in mitigating oxidative damage. In contrast, *B. napus* displayed lower activities of these enzymes, potentially reflecting a different adaptive strategy. This observed difference aligns with *B. napus*’s comparatively lower glucosinolate levels, indicating a potential trade-off between the activation of antioxidant enzymes and the accumulation of glucosinolates as part of their stress response mechanisms. Further investigations into these contrasting strategies may unveil insights into the nuanced interplay between antioxidant defence mechanisms and secondary metabolite accumulation in *Brassica* species under NaCl stress. 

The noted differences in antioxidant enzyme activities between *B. juncea* and *B. napus* under NaCl stress align well with our previous investigation into the photosynthetic performance of various *Brassica* species under similar conditions [90]. In our earlier study, we observed that *B. juncea* exhibited enhanced protection of the PSII reaction centers compared with *B. napus* under 300 mM NaCl conditions. This heightened protection probably contributed to the superior photosynthetic performance of *B. juncea* in response to stress. The interplay between antioxidant defence mechanisms, glucosinolate accumulation, and the preservation of PSII reaction centers underscores the complex adaptive strategies employed by different *Brassica* species to cope with saline stress.

The analysis of selected *GTR-*coding genes in different plant parts under stressful conditions revealed a divergent expression pattern in both species. The spatio-temporal expression of *GTR* genes elucidates their specific roles in particular organs or tissues. These findings align with previous studies [42,91,92] that reported varying *GTR* gene expression in different tissues of various *Brassica* species. Similar preferential gene expression in specific organs or tissues within the same gene family was noted in other studies [69,70,76]. Furthermore, variations in gene expression have been observed at different developmental stages within the same tissue, as reported by [93]. Our results are in line with earlier studies conducted on *GTR* in *B. oleracea* by [91]. In that study, the authors documented the expression of different *GTR* genes under NaCl stress conditions in different plant parts such as roots, hypocotyl, and leaves and observed varying expression patterns in the target tissues, as observed in the present study. 

Statistical modelling was employed to predict the impact of NaCl stress on the total glucosinolate content in *B. juncea* and *B. napus*. Results revealed contrasting effects, with an increase in glucosinolate content observed in *B. juncea* under escalating NaCl stress, aligning with findings in various *Brassica* species subjected to salinity stress [94,95,96]. This upsurge implies an elevated rate of secondary metabolism in *B. juncea*, potentially supporting plant survival under NaCl conditions. Conversely, *B. napus* exhibited a decreasing trend in total glucosinolate content, akin to observations in some sensitive *Brassica* species, suggesting the breakdown of these secondary metabolites due to NaCl stress [97]. It is noteworthy that the impact of salinity on total glucosinolate content is influenced by factors such as genetic background, environmental and soil conditions, developmental stage, plant part analyzed, and the applied salinity dose [98,99,100]. Additionally, under control conditions, *B. juncea* exhibited higher glucosinolate content compared with *B. napus* (Figure S2), highlighting the potential contribution of glucosinolates to *B. juncea*’s tolerance and established resilience to various biotic and abiotic factors in comparison to *B. napus*.

In summary, the divergent responses of glucosinolate content in *B. juncea* and *B. napus* under NaCl stress underscore the complex interplay between salinity, genetic factors, and environmental conditions. The observed increase in glucosinolates in *B. juncea* suggests a potential adaptive strategy, while the decrease in *B. napus* hints at a different response, likely influenced by species-specific sensitivities and stress-induced breakdown of secondary metabolites. The higher baseline glucosinolate content in *B. juncea* further underscores its resilience to diverse challenges, emphasizing the multifaceted role of glucosinolate in plant stress responses. 

## 5. Conclusions

The *GTR* gene family underwent expansion throughout its evolutionary trajectory from Arabidopsis to diploid and amphidiploid *Brassica* species. While structural and functional characteristics of *GTR* genes and proteins remained largely conserved in *Brassica* species, exceptions were also noted. Notably, *B. juncea* exhibited greater tolerance to NaCl stress compared with *B. napus*, as evidenced by biochemical and antioxidant assays. This tolerance in *B. juncea* was associated with its higher glucosinolate content and the augmentation of these compounds under NaCl stress, in contrast to *B. napus*. Furthermore, *GTR* genes displayed diverse expression patterns across different tissues and under varying stress conditions in both species.

Generally, the majority of *GTR* genes were upregulated under salt stress in *B. juncea*, suggesting active transport of glucosinolates to salt-stressed sites. Conversely, *B. napus* exhibited downregulation of most *GTR* genes under NaCl stress. Collectively, *B. juncea* emerges as the more salt-tolerant species relative to *B. napus*, characterized by lower oxidative stress parameters, heightened antioxidant activities, elevated glucosinolate content, and enhanced glucosinolate transport to stress sites via upregulation of *GTR* genes under NaCl stress conditions.

## Figures and Tables

**Table 1:** Pairwise sequence similarity and divergence between GTR proteins from Arabidopsis, *B. napus*, and *B. juncea*. Values in blue and grey colour indicate similarity and divergence, respectively. The intensity of the colour corresponds to the magnitude of the value.

		Similarity (%)																									
		AtGTR1	AtGTR2	BnaGTR1A1	BnaGTR1A2	BnaGTR1A3	BnaGTR1C1	BnaGTR1C2	BnaGTR1C3	BnaGTR2A1	BnaGTR2A2	BnaGTR2A3	BnaGTR2C1	BnaGTR2C2	BnaGTR2C3	BjuGTR1A1	BjuGTR1A2	BjuGTR1A3	BjuGTR1B1	BjuGTR1B2	BjuGTR1B3	BjuGTR2A1	BjuGTR2A2	BjuGTR2A3	BjuGTR2B1	BjuGTR2B2	BjuGTR2B3
**Divergence (%)**	AtGTR1		81.4	86.4	85.1	80.7	86.3	85.1	80.4	81.2	78.2	77.9	81.1	81.2	77.7	86.4	85.3	83.1	86.3	85.6	83.2	81.2	81.2	77.9	81.2	80.9	78.5
	AtGTR2	18.2		79.2	79.7	77.0	79.0	79.8	77.0	92.5	92.2	85.5	92.3	92.2	85.4	79.2	80.3	77.1	78.5	79.7	77.9	92.5	92.2	85.0	92.7	92.2	86.1
	BnaGTR1A1	12.9	19.6		89.7	88.0	99.2	89.9	87.7	80.3	79.8	77.2	80.2	79.8	78.3	99.8	90.2	88.4	96.9	89.9	88.4	80.5	79.8	77.5	80.0	80.0	78.3
	BnaGTR1A2	14.3	18.4	7.7		85.4	89.4	98.6	85.2	81.0	79.8	77.3	80.8	79.8	76.9	89.7	99.2	85.4	89.0	96.4	87.2	81.0	79.8	77.3	80.8	79.7	77.3
	BnaGTR1A3	17.3	21.8	10.3	12.0		87.9	85.5	97.5	77.5	77.8	74.0	77.3	77.8	74.5	88.0	85.4	99.7	88.9	85.4	94.5	77.6	77.8	75.3	77.1	77.5	74.8
	BnaGTR1C1	13.4	20.1	0.4	8.2	10.7		89.5	87.4	80.2	79.7	76.9	80.0	79.7	77.2	99.4	89.9	88.2	96.8	89.5	88.2	80.3	79.7	77.3	79.8	79.8	78.2
	BnaGTR1C2	14.5	18.4	7.7	1.2	12.0	8.2		85.4	81.0	79.8	77.1	80.8	79.8	76.9	89.9	98.9	85.5	89.2	98.9	87.2	81.0	79.8	77.3	80.8	79.7	77.3
	BnaGTR1C3	17.5	22.0	10.7	12.5	2.0	11.2	12.5		77.3	77.8	73.5	77.1	77.8	74.2	87.7	85.7	97.9	88.4	85.5	94.3	77.5	77.8	74.5	77.0	77.5	74.3
	BnaGTR2A1	18.0	6.7	17.7	17.0	21.1	18.2	17.0	21.5		94.8	88.4	99.8	94.8	88.2	80.3	81.3	77.6	79.5	81.6	78.4	99.8	94.8	86.9	99.8	94.6	89.9
	BnaGTR2A2	18.4	7.3	18.7	19.4	21.3	19.1	19.1	21.5	4.0		87.9	94.6	89.4	81.2	81.0	80.2	78.0	79.3	79.8	77.8	94.8	99.8	87.9	94.6	97.9	86.9
	BnaGTR2A3	23.0	13.8	22.5	21.8	25.5	22.8	21.8	26.3	11.2	11.0		88.2	87.9	97.7	77.2	77.4	74.2	76.6	77.9	74.9	88.6	87.9	96.2	88.4	88.1	94.1
	BnaGTR2C1	18.2	6.9	18.0	17.3	21.3	18.4	17.3	21.8	0.2	4.2	11.4		94.6	88.1	80.2	81.2	77.5	79.3	81.5	78.2	99.8	94.6	86.7	97.9	94.4	89.8
	BnaGTR2C2	18.7	7.3	18.9	19.4	21.3	19.4	19.1	21.5	4.0	0.2	11.0	4.2		87.7	79.8	80.2	78.0	79.3	81.0	78.2	94.8	89.4	86.9	94.2	97.7	89.4
	BnaGTR2C3	22.5	13.8	22.0	22.0	24.7	22.3	22.0	25.2	11.6	11.2	2.0	11.8	11.2		77.5	77.2	74.7	76.9	78.0	75.7	88.4	87.7	95.8	88.1	87.9	94.6
	BjuGTR1A1	12.9	19.6	0.0	7.7	10.3	0.4	7.7	10.7	17.7	18.7	22.5	18.0	18.9	22.0		90.2	88.4	97.1	89.9	88.4	80.5	79.8	77.5	80.0	80.0	78.3
	BjuGTR1A2	14.1	18.0	7.5	0.6	11.8	7.9	1.0	12.3	16.8	18.9	21.3	17.0	18.9	21.5	7.5		85.4	89.5	96.7	87.7	81.3	80.2	77.7	81.2	80.0	77.7
	BjuGTR1A3	17.0	21.5	10.1	11.8	0.2	10.5	11.8	1.8	20.8	21.1	25.2	21.1	21.1	24.5	10.1	11.6		89.2	85.4	94.8	77.8	78.0	75.3	77.3	77.6	74.9
	BjuGTR1B1	12.9	19.6	2.4	8.2	9.4	2.8	8.2	9.9	17.7	18.2	22.5	18.0	18.4	22.0	2.4	7.9	9.2		89.4	89.2	79.7	79.3	77.3	79.2	79.5	77.5
	BjuGTR1B2	14.1	19.4	8.2	3.2	12.5	8.6	3.4	12.5	16.6	18.0	21.1	16.8	18.0	20.8	8.2	3.2	12.3	8.4		87.4	81.6	81.0	77.9	81.6	81.0	78.3
	BjuGTR1B3	16.1	20.1	9.6	10.7	3.8	10.1	11.0	4.2	19.4	20.3	24.2	19.6	20.3	23.2	9.6	10.5	3.6	8.6	11.0		78.5	78.2	76.0	78.2	78.0	75.7
	BjuGTR2A1	18.0	6.7	17.7	17.0	21.1	18.2	17.0	21.5	0.0	4.0	11.2	0.2	4.0	11.6	17.7	16.8	20.8	17.7	16.6	19.4		94.8	86.9	98.0	94.6	90.1
	BjuGTR2A2	18.4	7.3	18.7	19.4	21.3	19.1	19.1	21.5	4.0	0.0	11.0	4.2	0.2	11.2	18.7	18.9	21.1	18.2	18.0	20.3	4.0		86.9	94.6	97.9	89.4
	BjuGTR2A3	24.7	16.3	25.0	24.2	27.3	25.2	24.2	28.3	14.1	13.8	4.0	14.3	13.8	4.4	25.0	23.7	27.3	25.0	23.5	26.3	14.1	13.8		87.3	87.3	91.7
	BjuGTR2B1	18.0	6.5	18.4	17.0	21.5	18.9	17.0	22.0	1.6	4.2	11.0	1.8	4.2	11.4	18.4	16.8	21.3	18.4	16.6	19.6	1.6	4.2	13.6		94.4	89.6
	BjuGTR2B2	18.4	7.5	18.2	19.1	21.1	18.7	18.9	21.3	4.8	1.2	11.0	5.0	1.4	11.2	18.2	18.7	20.8	17.7	17.5	19.8	4.8	1.2	13.6	5.0		89.4
	BjuGTR2B3	22.5	12.7	21.8	22.5	25.2	22.3	22.5	25.7	9.4	9.0	6.1	9.6	9.0	5.8	21.8	22.0	25.0	22.0	21.3	24.0	9.4	9.0	8.8	9.4	9.4	

**Table 2:** Statistical model to explain seed glucosinolates content.

Gene	Coefficients	Estimate	Standard Error	*t*-Value	Pr (>|*t*|)	Significance	Cumulative % Variance Explained
	(Intercept)	137.31	5.67	24.2	1.3 × 10^−14^	***	-
*GTR2A2* ^1^	Stem	21.46	7.84	2.73	0.0153	*	52.57
	Root	0.93	0.53	1.73	0.1027	—	60.52
*GTR2B1/C2* ^2^	Flower	14.73	3.69	3.98	0.0010	**	49.83
*GTR2A1* ^3^	Flower	19.01	8.50	2.23	0.0409	*	45.33
	Root	9.54	4.30	2.21	0.0425	*	58.82
*GTR1A2/B1* ^4^	Root	2.22	1.00	2.22	0.0417	*	47.17
	Stem	16.59	7.96	2.08	0.0548	—	59.02

^1^ Residual standard error: 16.1 on 15° of freedom, multiple R-squared: 0.6052, adjusted R-squared: 0.5526. ^2^ Residual standard error: 17.58 on 16 degrees of freedom, multiple R-squared: 0.4983, adjusted R-squared: 0.4669. ^3^ Residual standard error: 16.44 on 15 degrees of freedom, multiple R-squared: 0.5882, adjusted R-squared: 0.5333. ^4^ Residual standard error: 16.41 on 15 degrees of freedom, multiple R-squared: 0.5902, adjusted R-squared: 0.535. Significant differences are shown with different asterisks (***, **, * at *p* = 0.0001, 0.001, and 0.01, respectively).

## Data Availability

Data is contained within the article or Supplementary Material.

## Supplementary Materials

The following supporting information can be downloaded at: https://www.mdpi.com/article/10.3390/metabo14040179/s1, Figure S1: Multiple sequence alignment of GTR proteins from Arabidopsis, *B. juncea* and *B. napus*; Figure S2: Concentration of overall glucosinolates content in seed; Table S1: List of primers used for expression analysis in this study; Table S2; *GTR* genes and their respective protein properties; Table S3: K_a_, K_s_ and K_a_/K_s_ values of *GTR* gene pairs; Table S4: Mean squares and levels of significance from ANOVA for (a) biochemical parameters, (b) non-enzymatic antioxidants, (c) *GTR* genes and (d) glucosinolates content in *B. juncea* and *B. napus* under NaCl stress.

## References

[1] Cheng F., Wu J., Wang X. (2014). Genome triplication drove the diversification of *Brassica* plants. Hortic. Res..

[2] Cheng F., Sun R., Hou X., Zheng H., Zhang F., Zhang Y., Liu B., Liang J., Zhuang M., Liu Y. (2016). Subgenome parallel selection is associated with morphotype diversification and convergent crop domestication in *Brassica rapa* and *Brassica oleracea*. Nat. Genet..

[3] Augustine R., Arya G.C., Nambiar D.M., Kumar R., Bisht N.C. (2014). Translational genomics in *Brassica* crops: Challenges, progress, and future prospects. Plant Biotechnol. Rep..

[4] Nagaharu U., Nagaharu N. (1935). Genome analysis in *Brassica* with special reference to the experimental formation of *B. napus* and peculiar mode of fertilization. Jpn. J. Bot..

[5] Zandberg J.D., Fernandez C.T., Danilevicz M.F., Thomas W.J.W., Edwards D., Batley J. (2022). The Global Assessment of Oilseed *Brassica* Crop Species Yield, Yield Stability and the Underlying Genetics. Plants.

[6] Farooq N., Nawaz M.A., Mukhtar Z., Ali I., Hundleby P., Ahmad N. (2019). Investigating the *in vitro* regeneration potential of commercial cultivars of *Brassica*. Plants.

[7] Machado R.M.A., Serralheiro R.P. (2017). Soil salinity: Effect on vegetable crop growth. Management practices to prevent and mitigate soil salinization. Horticulturae.

[8] Jamil M., Lee K.B., Jung K.Y., Lee D.B., Han M.S., Rha E.S. (2007). Salt stress inhibits germination and early seedling growth in cabbage (*Brassica oleracea capitata* L.). Pak. J. Biol. Sci..

[9] Purty R.S., Kumar G., Singla-Pareek S.L., Pareek A. (2008). Towards salinity tolerance in *Brassica*: An overview. Physiol. Mol. Biol. Plants.

[10] Ghuge S.A., Rai A.N., Khandagale B.G., Penna S. (2011). Salt-induced stress responses of *Brassica* (*Brassica juncea* L.) genotypes. Arch. Agron. Soil Sci..

[11] Qasim M., Ashraf M., Ashraf M.Y., Rehman S.U., Rha E.S. (2003). Salt-induced changes in two canola cultivars differing in salt tolerance. Biol. Plant..

[12] Pang Q., Guo J., Chen S., Chen Y., Zhang L., Fei M., Jin S., Li M., Wang Y., Yan X. (2012). Effect of salt treatment on the glucosinolate-myrosinase system in *Thellungiella salsuginea*. Plant Soil.

[13] Ishida M., Hara M., Fukino N., Kakizaki T., Morimitsu Y. (2014). Glucosinolate metabolism, functionality and breeding for the improvement of *Brassicaceae* vegetables. Breed. Sci..

[14] Fahey J.W., Olson M.E., Stephenson K.K., Wade K.L., Chodur G.M., Odee D., Nouman W., Massiah M., Alt J., Egner P.A. (2018). The diversity of chemoprotective glucosinolates in *Moringaceae* (*Moringa* spp.). Sci. Rep..

[15] Annaz H., Sane Y., Bitchagno G.T.M., Ben Bakrim W., Drissi B., Mahdi I., El Bouhssini M., Sobeh M. (2022). Caper (*Capparis spinosa* L.): An updated review on its phytochemistry, nutritional value, traditional uses, and therapeutic potential. Front. Pharmacol..

[16] Jioe I.P.J., Lin H.-L., Shiesh C.-C. (2022). The investigation of phenylalanine, glucosinolate, benzylisothiocyanate (BITC) and cyanogenic glucoside of papaya fruits (*Carica papaya* L. cv.‘*Tainung No. 2*’) under different development stages between seasons and their correlation with bitter taste. Horticulturae.

[17] Halkier B.A., Gershenzon J. (2006). Biology and biochemistry of glucosinolates. Annu. Rev. Plant Biol..

[18] Hayes J.D., Kelleher M.O., Eggleston I.M. (2008). The cancer chemopreventive actions of phytochemicals derived from glucosinolates. Eur. J. Nutr.

[19] Dinkova-Kostova A.T., Kostov R.V. (2012). Glucosinolates and isothiocyanates in health and disease. Trends Mol. Med..

[20] Fahey J.W., Wehage S.L., Holtzclaw W.D., Kensler T.W., Egner P.A., Shapiro T.A., Talalay P. (2012). Protection of humans by plant glucosinolates: Efficiency of conversion of glucosinolates to isothiocyanates by the gastrointestinal microflora. Cancer Prev. Res..

[21] Mithen R.F., Dekker M., Verkerk R., Rabot S., Johnson I.T. (2000). The nutritional significance, biosynthesis and bioavailability of glucosinolates in human foods. J. Sci. Food Agric..

[22] Tanii H., Takayasu T., Higashi T., Leng S., Saijoh K. (2004). Allylnitrile: Generation from cruciferous vegetables and behavioral effects on mice of repeated exposure. Food Chem. Toxicol.

[23] Wallig M.A., Belyea R.L., Tumbleson M.E. (2002). Effect of pelleting on glucosinolate content of *Crambe* meal. Anim. Feed Sci. Technol..

[24] Tripathi M.K., Mishra A.S. (2007). Glucosinolates in animal nutrition: A review. Anim. Feed Sci. Technol..

[25] Augustine R., Mukhopadhyay A., Bisht N.C. (2013). Targeted silencing of *BjMYB28* transcription factor gene directs development of low glucosinolate lines in oilseed *Brassica juncea*. Plant Biotechnol. J..

[26] Mejicanos G., Sanjayan N., Kim I.H., Nyachoti C.M. (2016). Recent advances in canola meal utilization in swine nutrition. J. Anim. Sci. Technol..

[27] Cools K., Terry L.A. (2018). The effect of processing on the glucosinolate profile in mustard seed. Food Chem..

[28] Radojčić Redovniković I., Glivetić T., Delonga K., Vorkapić-Furač J. (2008). Glucosinolates and their potential role in plant. Period. Biol..

[29] Barth C., Jander G. (2006). *Arabidopsis* myrosinases *TGG1* and *TGG2* have redundant function in glucosinolate breakdown and insect defense. Plant J..

[30] Keum Y.-S., Jeong W.-S., Kong A.N.T. (2004). Chemoprevention by isothiocyanates and their underlying molecular signaling mechanisms. Mutat. Res.-Fund. Mol. Mech. Mutagen..

[31] Ellerbrock B.L.J., Kim J.H., Jander G. (2007). Contribution of glucosinolate transport to *Arabidopsis* defense responses. Plant Signal. Behav..

[32] Ahuja I., Rohloff J., Bones A.M. (2011). Defence mechanisms of *Brassicaceae*: Implications for plant-insect interactions and potential for integrated pest management. Sustain. Agric..

[33] Touw A.J., Verdecia Mogena A., Maedicke A., Sontowski R., Van Dam N.M., Tsunoda T. (2020). Both biosynthesis and transport are involved in glucosinolate accumulation during root-herbivory in *Brassica rapa*. Front. Plant Sci..

[34] Tierens K.F.M.J., Thomma B.P.H.J., Brouwer M., Schmidt J., Kistner K., Porzel A., Mauch-Mani B., Cammue B.P.A., Broekaert W.F. (2001). Study of the role of antimicrobial glucosinolate-derived isothiocyanates in resistance of *Arabidopsis* to microbial pathogens. Plant Physiol..

[35] Calmes B., N’Guyen G., Dumur J., Brisach C.A., Campion C., Iacomi B., Pigné S., Dias E., Macherel D., Guillemette T. (2015). Glucosinolate-derived isothiocyanates impact mitochondrial function in fungal cells and elicit an oxidative stress response necessary for growth recovery. Front. Plant Sci..

[36] Wittstock U., Kurzbach E., Herfurth A.M., Stauber E.J. (2016). Glucosinolate breakdown. Advances in Botanical Research.

[37] Abuyusuf M., Robin A.H.K., Lee J.-H., Jung H.-J., Kim H.-T., Park J.-I., Nou I.-S. (2018). Glucosinolate profiling and expression analysis of glucosinolate biosynthesis genes differentiate white mold resistant and susceptible cabbage lines. Int. J. Mol. Sci..

[38] Yan X., Chen S. (2007). Regulation of plant glucosinolate metabolism. Planta.

[39] Mann A., Kumari J., Kumar R., Kumar P., Pradhan A.K., Pental D., Bisht N.C. (2023). Targeted editing of multiple homologues of *GTR1* and *GTR2* genes provides the ideal low-seed, high-leaf glucosinolate oilseed mustard with uncompromised defence and yield. Plant Biotechnol. J..

[40] Nour-Eldin H.H., Andersen T.G., Burow M., Madsen S.R., Jørgensen M.E., Olsen C.E., Dreyer I., Hedrich R., Geiger D., Halkier B.A. (2012). *NRT/PTR* transporters are essential for translocation of glucosinolate defence compounds to seeds. Nature.

[41] Léran S., Varala K., Boyer J.-C., Chiurazzi M., Crawford N., Daniel-Vedele F., David L., Dickstein R., Fernandez E., Forde B. (2014). A unified nomenclature of *NITRATE TRANSPORTER 1/PEPTIDE TRANSPORTER* family members in plants. Trends Plant Sci..

[42] Nour-Eldin H.H., Madsen S.R., Engelen S., Jørgensen M.E., Olsen C.E., Andersen J.S., Seynnaeve D., Verhoye T., Fulawka R., Denolf P. (2017). Reduction of antinutritional glucosinolates in *Brassica* oilseeds by mutation of genes encoding transporters. Nat. Biotechnol..

[43] Nambiar D.M., Kumari J., Augustine R., Kumar P., Bajpai P.K., Bisht N.C. (2021). *GTR1* and *GTR2* transporters differentially regulate tissue-specific glucosinolate contents and defence responses in the oilseed crop *Brassica juncea*. Plant Cell Environ..

[44] Mahmood T., Hussain M., Mustafa H.S.B., Hasan E., Aftab M. (2017). Aari canola: Pakistan’s first ever canola quality and short duration mustard (*Brassica juncea* L.) cultivar resilient to climate change. Int. J. Biol. Pharm. Al. Sci.

[45] Sun B., Tian Y.-X., Chen Q., Zhang Y., Luo Y., Wang Y., Li M.-Y., Gong R.-G., Wang X.-R., Zhang F. (2019). Variations in the glucosinolates of the individual edible parts of three stem mustards (*Brassica juncea*). R. Soc. Open Sci..

[46] Cakmak I., Horst W.J. (1991). Effect of aluminium on lipid peroxidation, superoxide dismutase, catalase, and peroxidase activities in root tips of soybean (*Glycine max*). Physiol. Plant..

[47] Velikova V., Yordanov I., Edreva A. (2000). Oxidative stress and some antioxidant systems in acid rain-treated bean plants: Protective role of exogenous polyamines. Plant Sci..

[48] Hamilton P.B., Van Slyke D.D. (1943). Amino acid determination with ninhydrin. J. Biol. Chem..

[49] Bradford M.M. (1976). A rapid and sensitive method for the quantitation of microgram quantities of protein utilizing the principle of protein-dye binding. Anal. Biochem..

[50] Aguilar-Garcia C., Gavino G., Baragaño-Mosqueda M., Hevia P., Gavino V.C. (2007). Correlation of tocopherol, tocotrienol, γ-oryzanol and total polyphenol content in rice bran with different antioxidant capacity assays. Food Chem..

[51] Beauchamp C., Fridovich I. (1971). Superoxide dismutase: Improved assays and an assay applicable to acrylamide gels. Anal. Biochem..

[52] Chaoui A., Mazhoudi S., Ghorbal M.H., El Ferjani E. (1997). Cadmium and zinc induction of lipid peroxidation and effects on antioxidant enzyme activities in bean (*Phaseolus vulgaris* L.). Plant Sci..

[53] Aebi H. (1984). [13] Catalase in vitro. Methods in Enzymology.

[54] Nakano Y., Asada K. (1981). Hydrogen peroxide is scavenged by ascorbate-specific peroxidase in spinach chloroplasts. Plant Cell Physiol..

[55] Bennett E.J., Brignell C.J., Carion P.W.C., Cook S.M., Eastmond P.J., Teakle G.R., Hammond J.P., Love C., King G.J., Roberts J.A. (2017). Development of a statistical crop model to explain the relationship between seed yield and phenotypic diversity within the *Brassica napus* genepool. Agronomy.

[56] Lu S., Sturtevant D., Aziz M., Jin C., Li Q., Chapman K.D., Guo L. (2018). Spatial analysis of lipid metabolites and expressed genes reveals tissue-specific heterogeneity of lipid metabolism in high-and low-oil *Brassica napus* L. seeds. Plant J..

[57] Yang Y., Zhu K., Li H., Han S., Meng Q., Khan S.U., Fan C., Xie K., Zhou Y. (2018). Precise editing of *CLAVATA* genes in *Brassica napus* L. regulates multilocular silique development. Plant Biotechnol. J..

[58] Cartea M.E., Velasco P. (2008). Glucosinolates in *Brassica* foods: Bioavailability in food and significance for human health. Phytochem. Rev..

[59] Bell L., Oloyede O.O., Lignou S., Wagstaff C., Methven L. (2018). Taste and flavor perceptions of glucosinolates, isothiocyanates, and related compounds. Mol. Nutr. Food Res..

[60] Andersen T.G., Nour-Eldin H.H., Fuller V.L., Olsen C.E., Burow M., Halkier B.A. (2013). Integration of biosynthesis and long-distance transport establish organ-specific glucosinolate profiles in vegetative *Arabidopsis*. Plant Cell.

[61] O'Brien D. (2016). Canola: Good protein source for dairy cattle. Agric. Res..

[62] Lysak M.A., Koch M.A. (2011). Phylogeny, genome, and karyotype evolution of crucifers (*Brassicaceae*). Genetics and Genomics of the Brassicaceae.

[63] Arias T., Beilstein M.A., Tang M., McKain M.R., Pires J.C. (2014). Diversification times among *Brassica* (*Brassicaceae*) crops suggest hybrid formation after 20 million years of divergence. Am. J. Bot..

[64] Lukens L.N., Quijada P.A., Udall J., Pires J.C., Schranz M.E., Osborn T.C. (2004). Genome redundancy and plasticity within ancient and recent *Brassica* crop species. Biol. J. Linn. Soc..

[65] He J., He X., Chang P., Jiang H., Gong D., Sun Q. (2020). Genome-wide identification and characterization of *TCP* family genes in *Brassica juncea* var. tumida. PeerJ.

[66] Ahmad N., Fatima S., Mehmood M.A., Zaman Q.U., Atif R.M., Zhou W., Rahman M.-U., Gill R.A. (2023). Targeted genome editing in polyploids: Lessons from *Brassica*. Front. Plant Sci..

[67] Areej A., Nawaz H., Aslam I., Danial M., Qayyum Z., Rasool U.A., Asif J., Khalid A., Serfraz S., Saleem F. (2023). Investigation of *NLR* genes reveals divergent evolution on NLRome in diploid and polyploid species in genus *trifolium*. Genes.

[68] Verma D., Lakhanpal N., Singh K. (2019). Genome-wide identification and characterization of abiotic-stress responsive *SOD* (*superoxide dismutase*) gene family in *Brassica juncea* and *B. rapa*. BMC Genom..

[69] Lohani N., Babaei S., Singh M.B., Bhalla P.L. (2021). Genome-wide in silico identification and comparative analysis of *Dof* gene family in *Brassica napus*. Plants.

[70] Wang P., Zhao Z., Zhang Z., Cai Z., Liao J., Tan Q., Xiang M., Chang L., Xu D., Tian Q. (2021). Genome-wide identification and analysis of *NPR* family genes in *Brassica juncea* var. tumida. Gene.

[71] Afridi M., Ahmad K., Malik S.S., Rehman N., Yasin M., Khan S.M., Hussain A., Khan M.R. (2022). Genome-wide identification, phylogeny, and expression profiling analysis of shattering genes in rapeseed and mustard plants. J. Genet. Eng. Biotechnol..

[72] He J., Gu L., Tan Q., Wang Y., Hui F., He X., Chang P., Gong D., Sun Q. (2022). Genome-wide analysis and identification of the *PEBP* genes of *Brassica juncea* var. Tumida. BMC Genom..

[73] Ma L., Qi W., Bai J., Li H., Fang Y., Xu J., Xu Y., Zeng X., Pu Y., Wang W. (2022). Genome-Wide Identification and Analysis of the *Ascorbate Peroxidase* (*APX*) Gene Family of Winter Rapeseed (*Brassica rapa* L.) under Abiotic Stress. Front. Genet..

[74] Tyagi S., Sharma S., Taneja M., Kumar R., Sembi J.K., Upadhyay S.K. (2017). Superoxide dismutases in bread wheat (*Triticum aestivum* L.): Comprehensive characterization and expression analysis during development and, biotic and abiotic stresses. Agri. Gene.

[75] Wang W., Zhang X., Deng F., Yuan R., Shen F. (2017). Genome-wide characterization and expression analyses of *superoxide dismutase* (*SOD*) genes in *Gossypium hirsutum*. BMC Genom..

[76] Cai Z., Chen Y., Liao J., Wang D. (2020). Genome-wide identification and expression analysis of jasmonate ZIM domain gene family in tuber mustard (*Brassica juncea* var. tumida). PLoS ONE.

[77] Hölzl G., Rezaeva B.R., Kumlehn J., Dörmann P. (2023). Ablation of glucosinolate accumulation in the oil crop *Camelina sativa* by targeted mutagenesis of genes encoding the transporters *GTR1* and *GTR2* and regulators of biosynthesis *MYB28* and *MYB29*. Plant Biotechnol. J..

[78] Newstead S. (2015). Molecular insights into proton coupled peptide transport in the *PTR* family of oligopeptide transporters. Biochim. Biophys. Acta.

[79] Yan N. (2015). Structural biology of the major facilitator superfamily transporters. Annu. Rev. Biophys..

[80] Jørgensen M.E., Xu D., Crocoll C., Ernst H.A., Ramírez D., Motawia M.S., Olsen C.E., Mirza O., Nour-Eldin H.H., Halkier B.A. (2017). Origin and evolution of transporter substrate specificity within the *NPF* family. Elife.

[81] Yamaguchi-Shinozaki K., Shinozaki K. (2005). Organization of cis-acting regulatory elements in osmotic-and cold-stress-responsive promoters. Trends Plant Sci..

[82] Schmitz R.J., Grotewold E., Stam M. (2022). Cis-regulatory sequences in plants: Their importance, discovery, and future challenges. Plant Cell.

[83] Desikan R., A.-H.-Mackerness S., Hancock J.T., Neill S.J. (2001). Regulation of the *Arabidopsis* transcriptome by oxidative stress. Plant Physiol..

[84] Mikkelsen M.D., Petersen B.L., Glawischnig E., Jensen A.B., Andreasson E., Halkier B.A. (2003). Modulation of *CYP79* genes and glucosinolate profiles in *Arabidopsis* by defense signaling pathways. Plant Physiol..

[85] Miao H., Wang J., Cai C., Chang J., Zhao Y., Wang Q. (2017). Accumulation of glucosinolates in broccoli. Glucosinolates.

[86] Fiorillo A., Manai M., Visconti S., Camoni L. (2023). The Salt Tolerance–Related Protein (STRP) Is a Positive Regulator of the Response to Salt Stress in *Arabidopsis thaliana*. Plants.

[87] Narayani M., Srivastava S. (2017). Elicitation: A stimulation of stress in *in vitro* plant cell/tissue cultures for enhancement of secondary metabolite production. Phytochem. Rev..

[88] Rahman A., Albadrani G.M., Waraich E.A., Awan T.H., Yavaş İ., Hussain S. (2023). Plant Secondary Metabolites and Abiotic Stress Tolerance: Overview and Implications. Plant Abiotic Stress Responses and Tolerance Mechanisms.

[89] Chen Y.-z., Pang Q.-Y., He Y., Zhu N., Branstrom I., Yan X.-F., Chen S. (2012). Proteomics and metabolomics of *Arabidopsis* responses to perturbation of glucosinolate biosynthesis. Mol. Plant.

[90] Farooq N., Khan M.O., Ahmed M.Z., Fatima S., Nawaz M.A., Abideen Z., Nielsen B.L., Ahmad N. (2023). Salt-induced modulation of ion transport and psii photoprotection determine the salinity tolerance of amphidiploid *Brassicas*. Plants.

[91] Jiang D., Lei J., Cao B., Wu S., Chen G., Chen C. (2019). Molecular cloning and characterization of three glucosinolate transporter (*GTR*) genes from Chinese kale. Genes.

[92] Zhao Y., Chen Z., Chen J., Chen B., Tang W., Chen X., Lai Z., Guo R. (2021). Comparative transcriptomic analyses of glucosinolate metabolic genes during the formation of Chinese kale seeds. BMC Plant Biol..

[93] Li M., Xie F., Li Y., Gong L., Luo Y., Zhang Y., Chen Q., Wang Y., Lin Y., Zhang Y. (2020). Genome-wide analysis of the heat shock transcription factor gene family in *Brassica juncea*: Structure, evolution, and expression profiles. DNA Cell Biol..

[94] Arbona V., Manzi M., de Ollas C., Gómez-Cadenas A. (2013). Metabolomics as a tool to investigate abiotic stress tolerance in plants. Int. J. Mol. Sci..

[95] Petretto G.L., Urgeghe P.P., Massa D., Melito S. (2019). Effect of salinity (NaCl) on plant growth, nutrient content, and glucosinolate hydrolysis products trends in rocket genotypes. Plant Physiol. Biochem..

[96] Maina S., Ryu D.H., Cho J.Y., Jung D.S., Park J.-E., Nho C.W., Bakari G., Misinzo G., Jung J.H., Yang S.-H. (2021). Exposure to salinity and light spectra regulates glucosinolates, phenolics, and antioxidant capacity of *Brassica carinata* L. microgreens. Antioxidants.

[97] Sarikamiş G., Cakir A. (2017). Influence of salinity on aliphatic and indole glucosinolates in broccoli (*Brassica oleracea* var. italica). Appl. Ecol. Environ. Res..

[98] Velasco P., Cartea M.E., González C., Vilar M., Ordás A. (2007). Factors affecting the glucosinolate content of kale (*Brassica oleracea acephala* group). J. Agric. Food Chem..

[99] Zaghdoud C., Alcaraz-Lopez C., Mota-Cadenas C., Martínez-Ballesta M.d.C., Moreno D.A., Ferchichi A., Carvajal M. (2012). Differential responses of two broccoli (*Brassica oleracea* L. var *Italica*) cultivars to salinity and nutritional quality improvement. Sci. World J..

[100] Gyawali S., Parkin I.A.P., Steppuhn H., Buchwaldt M., Adhikari B., Wood R., Wall K., Buchwaldt L., Singh M., Bekkaoui D. (2019). Seedling, early vegetative, and adult plant growth of oilseed rapes (*Brassica napus* L.) under saline stress. Can. J. Plant Sci..

