# Supplementary material for: Studying Salt-Induced Shifts in Gene Expression Patterns of Glucosinolate Transporters and Glucosinolate Accumulation in Two Contrasting Brassica Species
Source: Metabolites. 2024 Mar 22;14(4):179. doi: 10.3390/metabo14040179 (PMC11052333; doi:10.3390/metabo14040179)
Supplement: Supplementary file 1 [file metabolites-14-00179-s001.zip › metabolites-2903361-supplementary.pdf]

Studying Salt-Induced Shifts in Gene Expression Patterns of Glucosinolate Transporters and Glucosinolate Accumulation in Two Contrasting *Brassica* Species

Samia Fatima [1], Muhammad Omar Khan [1], Nadia Iqbal [1], Muhammad Mudassar Iqbal [1], Huma Qamar [2,3], Muhammad Imtiaz [1], Penny Hundleby [4], Zhengyi Wei [5,*] and Niaz Ahmad [1,*]

[1] National Institute for Biotechnology and Genetic Engineering College (NIBGE-C), Pakistan Institute for Engineering and Applied Sciences (PIEAS), Faisalabad 38000, Pakistan; saminafatime@nibge.org (S.F.); muhammadkhan@nibge.org (M.O.K.); nadiaiqba@nibge.org (N.I.); muhammadiqbal@nibge.org (M.M.I.); m.imtiazpk92@nibge.org (M.I.)

[2] Oilseeds Research Institute, Ayub Agricultural Research Institute, Faisalabad 38000, Pakistan; huma.qamar@research.uwa.edu.au

[3] School of Biological Sciences, The University of Western Australia, Crawley, WA 6009, Australia

[4] Department of Crop Genetics, John Innes Centre, Norwich Research Park, Norwich NR4 7UH, UK; penny.hundleby@jic.ac.uk

[5] Maize Research Institute, Guangxi Academy of Agricultural Sciences, Nanning 530007, China

* Correspondence: weizy80@gxaas.net or weizy80@163.com (Z.W.); nahmad@nibge.org or niazbloch@yahoo.com (N.A.)

Table S1: List of primers used for expression analysis in this study

Sr No	Primer Name	Sequence (5'— 3')
1	GTR2A2-F	GATTGCAACGGTACACAGGA
2	GTR2A2-R	CCATCTTGACATCACCAAGAA
3	GTR2B1/C2-F	CACAGGACGACGCAGAACT
4	GTR2B1/C2-R	AGCAAAATTAACCGCCAAGA
5	GTR2A1-F	TGATTGCAATGGTTCACAGG
6	GTR2A1-R	TTCCAGCAATCATGAAATAGAA
7	GTR1B1/A2-F	GAGCGGGGATCTTCTTTG
8	GTR1B1/A2-R	TCCATCCCTAGAGTCTGTTT
9	ACT-F	TGTGACAATGGAACTGGAAT
10	ACT-R	GACCCATCCCAACCATGA

Table S2: GTR genes and their respective protein properties. The table includes Gene IDs, Location of the gene on the chromosomes, transcript and protein length, molecular weight (Da), isoelectronic point (pI), grand average of hydropathy values (GRAVY) and sub-cellular localization of proteins.

Gene ID	Gene Name	Chr. No.	Strand	Location	Transcript length	Protein length	Mol. Weight (Da)	pI	GRAVY	Sub-cellular localization
AT3G47960.1	*AtGTR1*	3	-	17697942-17700833	1911	636	71039.51	9.11	0.197	Plasma membrane
BolC3t18961H	*BolGTR1C1*	C3	+	45537027-45539805	1905	634	70460.97	8.71	0.247	Plasma membrane
BolC1t03184H	*BolGTR1C2*	C1	+	24730858-24733298	1926	641	71173.45	8.96	0.127	Plasma membrane
BolC8t51846H	*BolGTR1C3*	C8	-	45270014-45272334	1764	587	64966.96	9.22	0.282	Plasma membrane
BniB08g061180.2N	*BniGTR1B1*	B8	-	57886043 -57888835	1848	615	68205.34	8.61	0.239	Plasma membrane
BniB06g024230.2N	*BniGTR1B2*	B6	+	14337898-14340309	1905	634	70522.05	8.71	0.268	Plasma membrane
BniB05g052360.2N	*BniGTR1B3*	B5	-	44394313 -44396700	1854	617	68368.62	8.86	0.232	Plasma membrane
BraA06g019540.3.5C	*BraGTR1A1*	A6	+	10801141 -10804360	1848	615	68053.42	8.99	0.276	Plasma membrane
BraA06g025210.3.5C	*BraGTR1A2*	A6	-	16715892-16718716	1905	634	70567.02	8.79	0.211	Plasma membrane
BraA01g025200.3.5C	*BraGTR1A3*	A1	+	14570408- 14573089	1815	604	67112.62	9.33	0.307	Plasma membrane
A06p19700.1_BnaDAR	*BnaGTR1A1*	A6	+	11067707 -11070559	1848	615	68069.42	8.99	0.272	Plasma membrane
A06p27390.1_BnaDAR	*BnaGTR1A2*	A6	-	32634402- 32637103	1905	634	70552.05	8.86	0.22	Plasma membrane
A01p25730.1_BnaDAR	*BnaGTR1A3*	A1	+	14825160- 14827590	1926	641	71245.69	9.02	0.152	Plasma membrane
C03p72210.1_BnaDAR	*BnaGTR1C1*	C3	-	55171571 -55174464	1848	615	68172.52	8.92	0.254	Plasma membrane
C03p66900.1_BnaDAR	*BnaGTR1C2*	C3	+	45114907 -45117916	1905	634	70460.97	8.71	0.247	Plasma membrane
C01p32480.1_BnaDAR	*BnaGTR1C3*	C1	+	24863749 -24866191	1926	641	71310.64	8.9	0.13	Plasma membrane
BjuVA06G19500	*BjuGTR1A1*	A6	+	10676591 - 10679465	1848	615	68127.46	8.92	0.263	Plasma membrane
BjuVA06G26150	*BjuGTR1A2*	A6	-	28729916-28732684	1905	634	70567.02	8.79	0.211	Plasma membrane
BjuVA01G26580	*BjuGTR1A3*	A1	+	14804496- 14806848	1848	615	68053.21	9	0.228	Plasma membrane
BjuVB08G47510	*BjuGTR1B1*	B8	-	53659983-53663145	1848	615	68205.34	8.61	0.239	Plasma membrane
BjuVB06G23560	*BjuGTR1B2*	B6	+	15676902-15679502	1905	634	70544.01	8.61	0.26	Plasma membrane
BjuVB05G42900	*BjuGTR1B3*	B5	+	31049142-31051530	1854	617	68340.53	8.8	0.233	Plasma membrane
BcaB01g00859	*BcaGTR1B1*	B1	+	7365410-7368203	1848	615	68205.34	8.61	0.239	Plasma membrane
BcaB02g10681	*BcaGTR1B2*	B2	+	30683181-30685582	1905	634	70544.01	8.61	0.26	Plasma membrane
BcaB05g23096	*BcaGTR1B3*	B5	-	16137173-16139533	1854	617	68402.68	8.92	0.236	Plasma membrane
BcaC01g01757	*BcaGTR1C1*	C1	+	18324250 -18326854	1848	615	68226.55	8.86	0.253	Plasma membrane
BcaC01g02298	*BcaGTR1C2*	C1	-	29981307-29984085	1905	634	70460.97	8.71	0.247	Plasma membrane
BcaC09g49832	*BcaGTR1C3*	C9	-	25815759- 25818111	1836	611	67635.63	8.89	0.224	Plasma membrane
AT5G62680.1	*AtGTR2*	5	-	25165137-25167943	1851	616	67890.98	9.1	0.284	Plasma membrane
BolC2t12292H	*BolGTR2C1*	C2	-	61815970 -61818365	1839	612	67626.82	8.92	0.333	Plasma membrane
BolC3t18666H	*BolGTR2C2*	C3	+	42224839 -42227233	1839	612	67725.12	8.98	0.335	Plasma membrane
BolC9t53791H	*BolGTR2C3*	C9	-	5348144-5350524	1839	612	67447.53	8.87	0.348	Plasma membrane
BniB04g002130.2N	*BniGTR2B1*	B4	+	1022088 -1024566	1839	612	67716.02	9.01	0.329	Plasma membrane
BniB06g010940.2N	*BniGTR2B2*	B6	-	5574061-5576460	1839	612	67786.23	9.1	0.331	Plasma membrane
BniB07g041760.2N	*BniGTR2B3*	B7	-	47577010 -47579359	1821	606	66729.74	8.78	0.335	Plasma membrane

BraA02g045700.3.5C	*BraGTR2A1*	A2	-	30298054-30300823	1839	612	67652.94	8.98	0.34	Plasma membrane
BraA06g027380.3.5C	*BraGTR2A2*	A6	-	18424905 -18427697	1839	612	67739.14	8.98	0.335	Plasma membrane
BraA09g007530.3.5C	*BraGTR2A3*	A9	-	4272215 -4274974	1839	612	67644.82	8.79	0.334	Plasma membrane
A02p42460.1_BnaDAR	*BnaGTR2A1*	A2	-	32205553 -32208391	1839	612	67640.89	8.98	0.332	Plasma membrane
A06p29420.1_BnaDAR	*BnaGTR2A2*	A6	-	34287866 -34290565	1839	612	67797.18	8.91	0.33	Plasma membrane
A09p07810.1_BnaDAR	*BnaGTR2A3*	A9	-	4463087 -4465822	1839	612	67644.82	8.79	0.334	Plasma membrane
C02p62380.1_BnaDAR	*BnaGTR2C1*	C2	-	60482227 -60490459	1839	612	67626.82	8.92	0.333	Plasma membrane
C03p64110.1_BnaDAR	*BnaGTR2C2*	C3	+	41808405 -41811139	1839	612	67725.12	8.98	0.335	Plasma membrane
C09p08780.1_BnaDAR	*BnaGTR2C3*	C9	-	5199813 -5202640	1836	611	67378.42	8.86	0.137	Plasma membrane
BjuVA02G47610	*BjuGTR2A1*	A2	-	31650239-31652620	1839	612	67656.89	8.98	0.328	Plasma membrane
BjuVA06G28410	*BjuGTR2A2*	A6	-	30414065-30416809	1839	612	67797.18	8.91	0.33	Plasma membrane
BjuVA09G07610	*BjuGTR2A3*	A9	-	4182819- 4194805	1581	526	57986.02	9	0.452	Plasma membrane
BjuVB04G01960	*BjuGTR2B1*	B4	+	1112996-1115472	1839	612	67625.89	9.02	0.326	Plasma membrane
BjuVB06G11810	*BjuGTR2B2*	B6	-	6701332-6704576	1839	612	67786.23	9.1	0.331	Plasma membrane
BjuVB07G18470	*BjuGTR2B3*	B7	+	10890421-10893018	1821	606	66694.69	8.78	0.333	Plasma membrane
BcaB02g09673	*BcaGTR2B1*	B2	-	21952429 -21954836	1839	612	67758.17	9.1	0.327	Plasma membrane
BcaNung01385	*BcaGTR2B2*	Contig503	+	38887-41234	1821	606	66694.69	8.78	0.333	Plasma membrane
BcaB07g29850	*BcaGTR2B3*	B7	-	1225649-1228114	1776	591	65345.13	8.64	0.361	Plasma membrane
BcaC01g02504	*BcaGTR2C1*	C1	-	32914562 -32924437	1836	611	67586.9	8.91	0.343	Plasma membrane
BcaC03g18168	*BcaGTR2C2*	C3	-	63135183-63154915	2223	740	81415.64	9.54	0.106	Plasma membrane
BcaC04g23397	*BcaGTR2C3*	C4	+	59044137-59046512	1836	611	67391.42	8.78	0.329	Plasma membrane

Table S3: K_a, K_s and K_a/K_s values of the *GTR* gene pairs

GTR 1	***GTR 2***	**K_a**	**K_s**	**K_a/K_s**
BnaGTR1A1	BjuGTR1A1	0.0007	0.0138	0.0514
BnaGTR1A3	BjuGTR1A3	0.0014	0.0068	0.2110
BnaGTR1C2	BjuGTR1A2	0.0052	0.0467	0.1112
BnaGTR2A1	BnaGTR2C1	0.0007	0.0762	0.0094
BnaGTR2A2	BnaGTR2C2	0.0007	0.0712	0.0100
BnaGTR2A3	BjuGTR2A3	0.0294	0.0638	0.4610

Table S4: Mean squares and levels of significance from ANOVA for (a) biochemical parameters, (b) non-enzymatic antioxidants, (c) GTR genes and (d) glucosinolates in *B. juncea* and *B. napus* under NaCl stress. (***, **, * at p = 0.0001, 0.001 and 0.01, respectively).

a) Response: Biochemical parameters

**Source of variation**	**DF**	**H_2O_2**	**MDA**	**Phenolics**	**TFA**	**TSP**
Species (Sp)	1	21.3942***	26.731*	0.0000445	0.00103397***	3.8068***
NaCl	2	6.4412***	77.217***	0.0038252***	0.00201673***	24.6687***
Sp x NaCl	2	0.4565*	0.873	0.0001121	0.00033979***	2.19***
Error	12	0.0972	2.902	0.0002278	0.00000442	0.0959

b) Response: Non-enzymatic antioxidants

**Source of variation**	**DF**	**POD**	**CAT**	**APX**	**SOD**
Species (Sp)	1	0.0001846*	18.76***	0.0047261***	2.041
NaCl	2	0.00197793***	4.3932*	0.0014783*	187.963***
Sp x NaCl	2	0.00021392*	0.9233g	0.0001319	21.086
Error	12	0.00003854	0.7102	0.0002205	7.936

c) Response: GTR genes

**Source of variations**	**DF**	**GTR1A2/B1**	**GTR2A2**	**GTR2A1**	**GTR2B1/C2**
Species (Sp)	1	53.125***	2.134	38.268·	37.270**
Plant part (PP)	4	34.32***	146.603**	27.604*	35.596***
Sp x PP	4	41.097***	272.908***	52.086***	65.552***
Error	80	2.457	34.9	10.121	4.966

d) Response: Glucosinolates

**Source of variations**	**DF**	**Sum Sq**	**Mean Sq**	***F* - Value**	**Pr (> *F*)**	**Significance**
Species	1	37868	37868	1900.880	2×10^{-16}	***
NaCl	2	180	90	4.509	0.0145	*
Residuals	68	1355	20	—	—	—

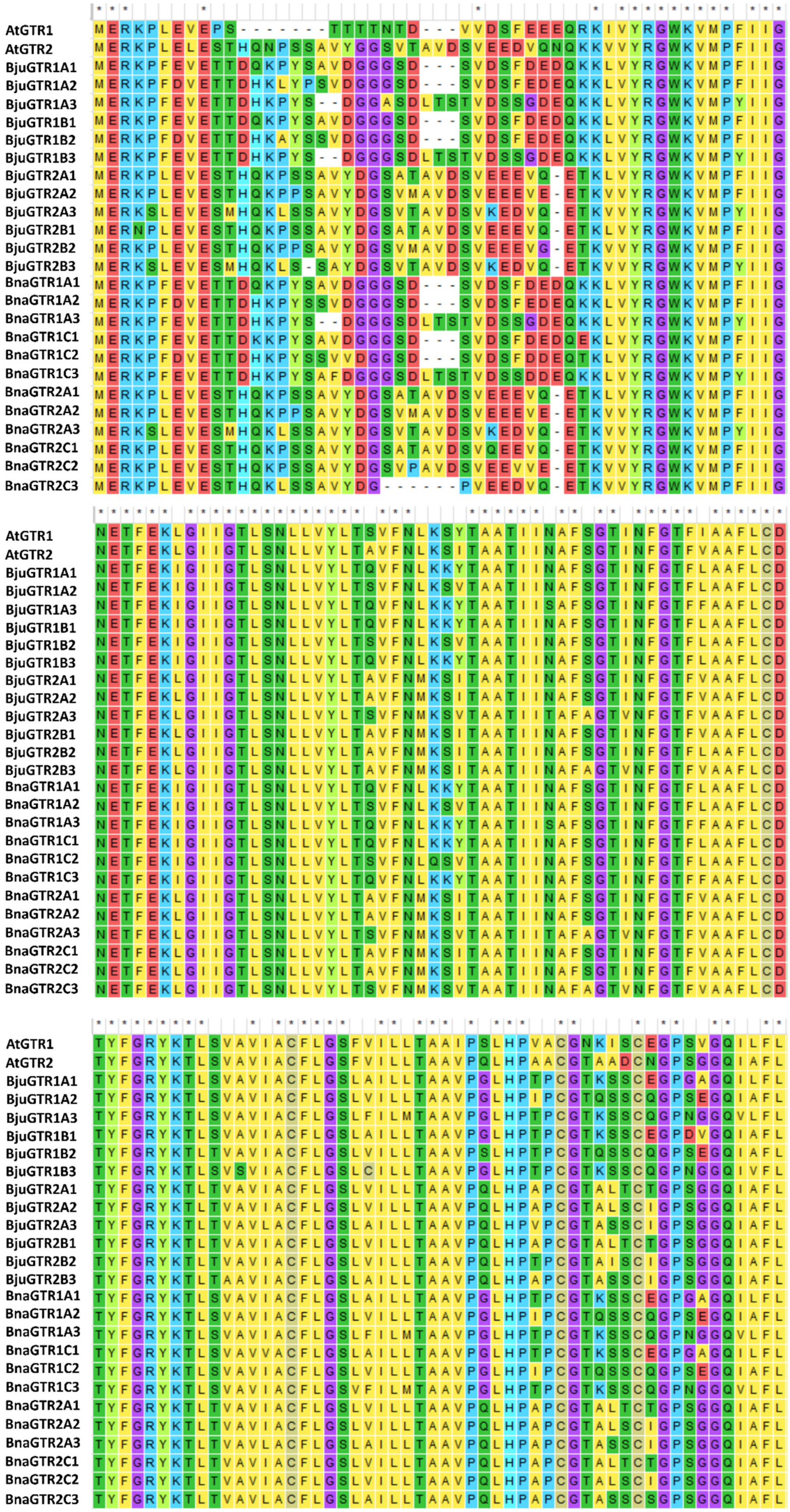

AtGTR1 MERKPLEVEPS-------TTTTNTD---VVDSFEEEQRKIVYRGWKVMPFIIG
AtGTR2 MERKPLELESTHQNPSSAVYGGSVTAVDSVEEDVQNQKKVVYRGWKVMPFIIG
BjuGTR1A1 MERKPFEVETTDQKPYSAVDGGGSD---SVDSFDEDQKKLVYRGWKVMPFIIG
BjuGTR1A2 MERKPFDVETTDHKLYPSVDGGGSD---SVDSFEDEQKKLVYRGWKVMPFIIG
BjuGTR1A3 MERKPFEVETTDHKPYS--DGGASDLTSTVDSSGDEQKKLVYRGWKVMPYIIG
BjuGTR1B1 MERKPFEVETTDQKPYSAVDGGGSD---SVDSFDEDQKKLVYRGWKVMPFIIG
BjuGTR1B2 MERKPFDVETTDHKAYSSVDGGGSD---SVDSFEDEQKKLVYRGWKVMPFIIG
BjuGTR1B3 MERKPFEVETTDHKPYS--DGGGSDLTSTVDSSGDEQKKLVYRGWKVMPYIIG
BjuGTR2A1 MERKPLEVESTHQKPSSAVYDGSATAVDSVEEEVQ-ETKLVYRGWKVMPFIIG
BjuGTR2A2 MERKPLEVESTHQKPPSAVYDGSVMAVDSVEEEVE-ETKVVYRGWKVMPFIIG
BjuGTR2A3 MERKSLEVESMHQKLSSAVYDGSVTAVDSVKEDVQ-ETKVVYRGWKVMPYIIG
BjuGTR2B1 MERNPLEVESTHQKPSSAVYDGSATAVDSVEEEVQ-ETKLVYRGWKVMPFIIG
BjuGTR2B2 MERKPLEVESTHQKPPSAVYDGSVMAVDSVEEEVG-ETKVVYRGWKVMPFIIG
BjuGTR2B3 MERKSLEVESMHQKLS-SAYDGSVTAVDSVKEDVQ-ETKVVYRGWKVMPYIIG
BnaGTR1A1 MERKPFEVETTDQKPYSAVDGGGSD---SVDSFDEDQKKLVYRGWKVMPFIIG
BnaGTR1A2 MERKPFDVETTDHKPYSSVDGGGSD---SVDSFEDEQKKLVYRGWKVMPFIIG
BnaGTR1A3 MERKPFEVETTDHKPYS--DGGGSDLTSTVDSSGDEQKKLVYRGWKVMPYIIG
BnaGTR1C1 MERKPFEVETTDKKPYSAVDGGGSD---SVDSFDEDQEKLVYRGWKVMPFIIG
BnaGTR1C2 MERKPFDVETTDHKPYSSVVDGGSD---SVDSFDDEQTKLVYRGWKVMPFIIG
BnaGTR1C3 MERKPFEVETTDHKPYSAFDGGGSDLTSTVDSSDDEQKKLVYRGWKVMPYIIG
BnaGTR2A1 MERKPLEVESTHQKPSSAVYDGSATAVDSVEEEVQ-ETKLVYRGWKVMPFIIG
BnaGTR2A2 MERKPLEVESTHQKPPSAVYDGSVMAVDSVEEEVE-ETKVVYRGWKVMPFIIG
BnaGTR2A3 MERKSLEVESMHQKLSSAVYDGSVTAVDSVKEDVQ-ETKVVYRGWKVMPYIIG
BnaGTR2C1 MERKPLEVESTHQKPSSAVYDGSATAVDSVQEEVQ-ETKVVYRGWKVMPFIIG
BnaGTR2C2 MERKPLEVESTHQKPSSAVYDGSVPAVDSVEEVVE-ETKVVYRGWKVMPFIIG
BnaGTR2C3 MERKPLEVESTHQKLSSAVYDG------PVEEDVQ-ETKVVYRGWKVMPFIIG
AtGTR1 NETFEKLGIIGTLSNLLVYLTSVFNLKSYTAATIINAFSGTINFGTFIAAFLCD
AtGTR2 NETFEKLGIIGTLSNLLVYLTAVFNLKSITAATIINAFSGTINFGTFVAAFLCD
BjuGTR1A1 NETFEKIGIIGTLSNLLVYLTQVFNLKKYTAATIINAFSGTINFGTFLAAFLCD
BjuGTR1A2 NETFEKIGIIGTLSNLLVYLTSVFNLKSVTAATIINAFSGTINFGTFLAAFLCD
BjuGTR1A3 NETFEKIGIIGTLSNLLVYLTQVFNLKKYTAATIISAFSGTINFGTFFAAFLCD
BjuGTR1B1 NETFEKIGIIGTLSNLLVYLTQVFNLKKYTAATIINAFSGTINFGTFLAAFLCD
BjuGTR1B2 NETFEKIGIIGTLSNLLVYLTSVFNLKSVTAATIINAFSGTINFGTFLAAFLCD
BjuGTR1B3 NETFEKIGIIGTLSNLLVYLTQVFNLKKYTAATIINAFSGTINFGTFLAAFLCD
BjuGTR2A1 NETFEKLGIIGTLSNLLVYLTAVFNMKSITAATIINAFSGTINFGTFVAAFLCD
BjuGTR2A2 NETFEKLGIIGTLSNLLVYLTAVFNMKSITAATIINAFSGTINFGTFVAAFLCD
BjuGTR2A3 NETFEKLGIIGTLSNLLVYLTSVFNMKSVTAATIITAFAGTVNFGTFVAAFLCD
BjuGTR2B1 NETFEKLGIIGTLSNLLVYLTAVFNMKSITAATIINAFSGTINFGTFVAAFLCD
BjuGTR2B2 NETFEKLGIIGTLSNLLVYLTAVFNMKSITAATIINAFSGTINFGTFLAAFLCD
BjuGTR2B3 NETFEKLGIIGTLSNLLVYLTAVFNMKSITAATIINAFAGTVNFGTFVAAFLCD
BnaGTR1A1 NETFEKIGIIGTLSNLLVYLTQVFNLKKYTAATIINAFSGTINFGTFLAAFLCD
BnaGTR1A2 NETFEKIGIIGTLSNLLVYLTSVFNLKSVTAATIINAFSGTINFGTFLAAFLCD
BnaGTR1A3 NETFEKIGIIGTLSNLLVYLTQVFNLKKYTAATIISAFSGTINFGTFFAAFLCD
BnaGTR1C1 NETFEKIGIIGTLSNLLVYLTQVFNLKKYTAATIINAFSGTINFGTFLAAFLCD
BnaGTR1C2 NETFEKIGIIGTLSNLLVYLTSVFNLQSVTAATIINAFSGTINFGTFLAAFLCD
BnaGTR1C3 NETFEKIGIIGTLSNLLVYLTQVFNLKKYTAATIINAFSGTINFGTFFAAFLCD
BnaGTR2A1 NETFEKLGIIGTLSNLLVYLTAVFNMKSITAATIINAFSGTINFGTFVAAFLCD
BnaGTR2A2 NETFEKLGIIGTLSNLLVYLTAVFNMKSITAATIINAFSGTINFGTFVAAFLCD
BnaGTR2A3 NETFEKLGIIGTLSNLLVYLTSVFNMKSVTAATIITAFAGTVNFGTFVAAFLCD
BnaGTR2C1 NETFEKLGIIGTLSNLLVYLTAVFNMKSITAATIINAFSGTINFGTFVAAFLCD
BnaGTR2C2 NETFEKLGIIGTLSNLLVYLTAVFNMKSITAATIINAFSGTINFGTFVAAFLCD
BnaGTR2C3 NETFEKLGIIGTLSNLLVYLTSVFNMKSVTAATIINAFAGTVNFGTFVAAFLCD
AtGTR1 TYFGRYKTLSVAVIACFLGSFVILLTAAIPSLHPVACGNKISCEGPSVGQILFL
AtGTR2 TYFGRYKTLSVAVIACFLGSFVILLTAAVPQLHPAACGTAADCNGPSGGQIAFL
BjuGTR1A1 TYFGRYKTLSVAVIACFLGSLAILLTAAVPGLHPTPCGTKSSCEGPGAGQILFL
BjuGTR1A2 TYFGRYKTLSVAVIACFLGSLVILLTAAVPGLHPIPCGTQSSCQGPSEGQIAFL
BjuGTR1A3 TYFGRYKTLSVAVIACFLGSLFILMTAAVPGLHPTPCGTKSSCQGPNGGQVLFL
BjuGTR1B1 TYFGRYKTLSVAVIACFLGSLAILLTAAVPGLHPTPCGTKSSCEGPDVGQIAFL
BjuGTR1B2 TYFGRYKTLTVAVIACFLGSLVILLTAAVPSLHPTPCGTQSSCQGPSEGQIAFL
BjuGTR1B3 TYFGRYKTLSVSVIACFLGSLCILLTAAVPGLHPTPCGTKSSCQGPNGGQIVFL
BjuGTR2A1 TYFGRYKTLTVAVIACFLGSLVILLTAAVPQLHPAPCGTALTCTGPSGGQIAFL
BjuGTR2A2 TYFGRYKTLTVAVIACFLGSLVILLTAAVPQLHPAPCGTALSCIGPSGGQIAFL
BjuGTR2A3 TYFGRYKTLTVAVLACFLGSLAILLTAAVPQLHPVPCGTASSCIGPSGGQIAFL
BjuGTR2B1 TYFGRYKTLTVAVIACFLGSLVILLTAAVPQLHPAPCGTALTCTGPSGGQIAFL
BjuGTR2B2 TYFGRYKTLTVAVIACFLGSLVILLTAAVPQLHPTPCGTAISCIGPSGGQIAFL
BjuGTR2B3 TYFGRYKTLTAAVIACFLGSLAILLTAAVPQLHPAPCGTASSCIGPSGGQIAFL
BnaGTR1A1 TYFGRYKTLSVAVIACFLGSLAILLTAAVPGLHPTPCGTKSSCEGPGAGQILFL
BnaGTR1A2 TYFGRYKTLSVAVIACFLGSLVILLTAAVPGLHPIPCGTQSSCQGPSEGQIAFL
BnaGTR1A3 TYFGRYKTLSVAVIACFLGSLFILMTAAVPGLHPTPCGTKSSCQGPNGGQVLFL
BnaGTR1C1 TYFGRYKTLSVAVVACFLGSLAILLTAAVPGLHPTPCGTKSSCEGPGAGQILFL
BnaGTR1C2 TYFGRYKTLSVAVIACFLGSLVILLTAAVPGLHPIPCGTQSSCQGPSEGQIAFL
BnaGTR1C3 TYFGRYKTLSVAVIACFLGSVFILMTAAVPGLHPTPCGTKSSCQGPNGGQVLFL
BnaGTR2A1 TYFGRYKTLTVAVIACFLGSLVILLTAAVPQLHPAPCGTALTCTGPSGGQIAFL
BnaGTR2A2 TYFGRYKTLTVAVIACFLGSLVILLTAAVPQLHPAPCGTALSCIGPSGGQIAFL
BnaGTR2A3 TYFGRYKTLTVAVLACFLGSLAILLTAAVPQLHPAPCGTASSCIGPSGGQIAFL
BnaGTR2C1 TYFGRYKTLTVAVIACFLGSLVILLTAAVPQLHPAPCGTALTCTGPSGGQIAFL
BnaGTR2C2 TYFGRYKTLTVAVIACFLGSLVILLTAAVPQLHPAPCGTALSCIGPSGGQIAFL
BnaGTR2C3 TYFGRYKTLTVAVLACFLGSLAILLTAAVPQLHPAPCGTASSCSGPSGGQIAFL

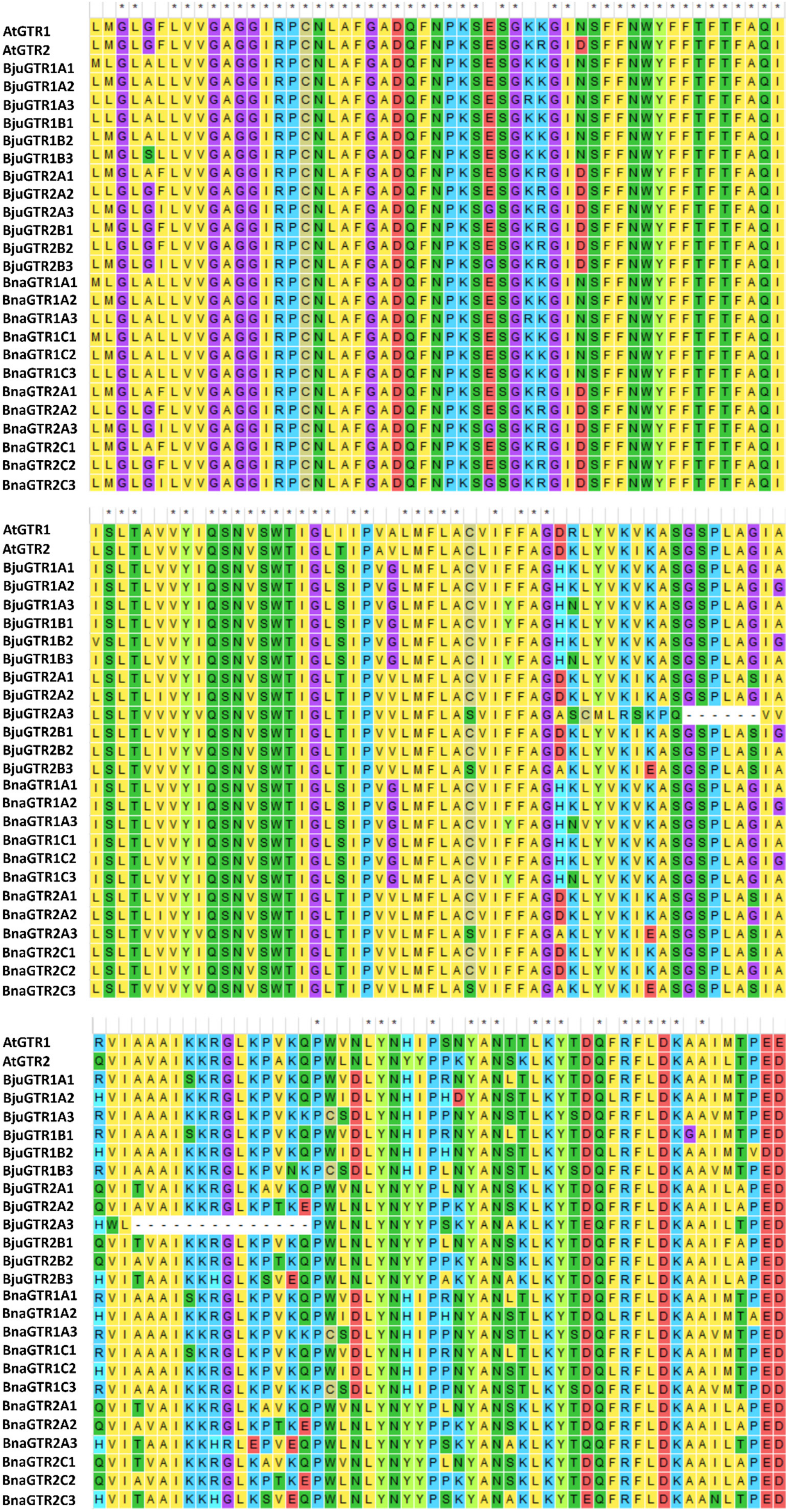
AtGTR1
AtGTR2
BjuGTR1A1
BjuGTR1A2
BjuGTR1A3
BjuGTR1B1
BjuGTR1B2
BjuGTR1B3
BjuGTR2A1
BjuGTR2A2
BjuGTR2A3
BjuGTR2B1
BjuGTR2B2
BjuGTR2B3
BnaGTR1A1
BnaGTR1A2
BnaGTR1A3
BnaGTR1C1
BnaGTR1C2
BnaGTR1C3
BnaGTR2A1
BnaGTR2A2
BnaGTR2A3
BnaGTR2C1
BnaGTR2C2
BnaGTR2C3

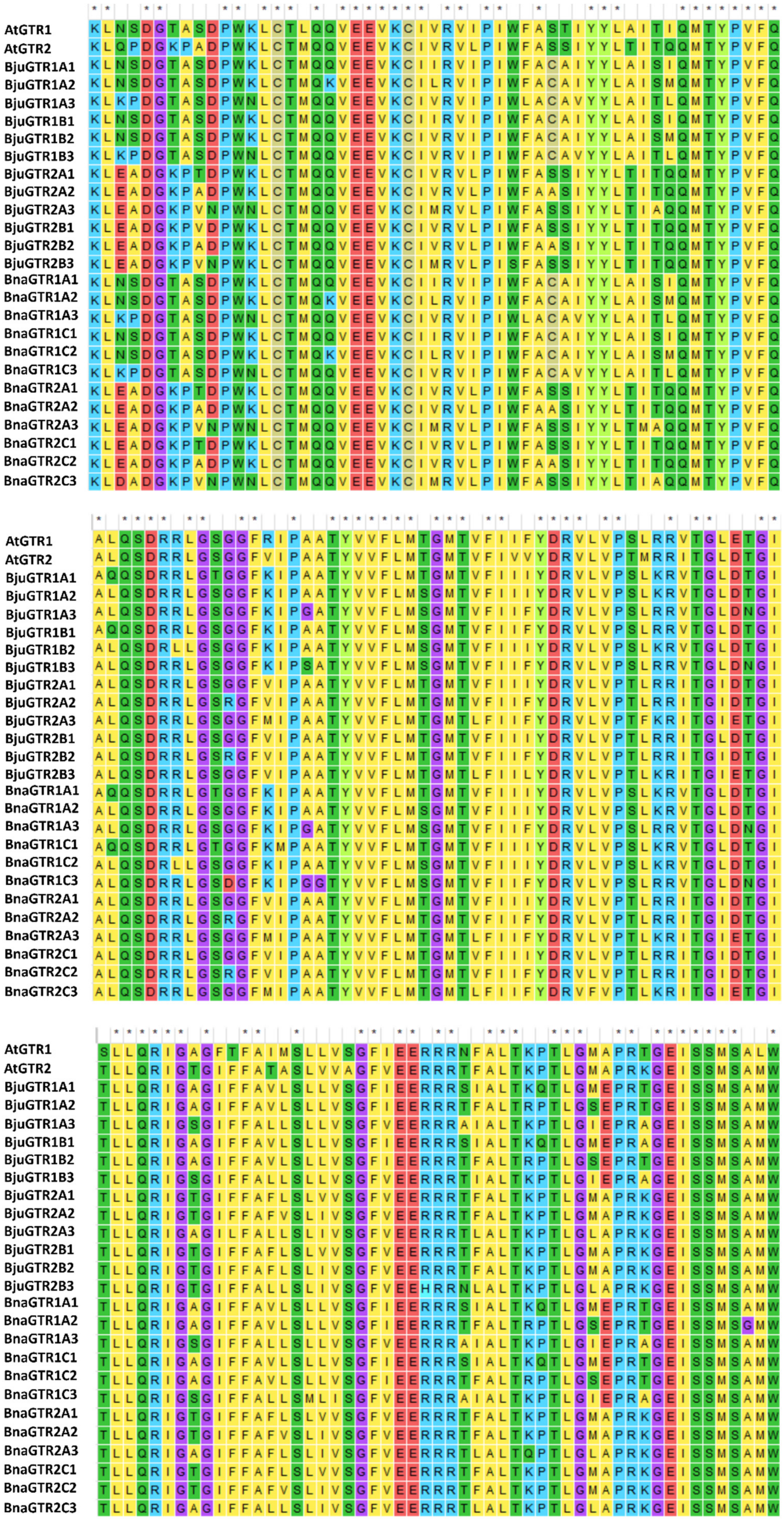
AtGTR1
AtGTR2
BjuGTR1A1
BjuGTR1A2
BjuGTR1A3
BjuGTR1B1
BjuGTR1B2
BjuGTR1B3
BjuGTR2A1
BjuGTR2A2
BjuGTR2A3
BjuGTR2B1
BjuGTR2B2
BjuGTR2B3
BnaGTR1A1
BnaGTR1A2
BnaGTR1A3
BnaGTR1C1
BnaGTR1C2
BnaGTR1C3
BnaGTR2A1
BnaGTR2A2
BnaGTR2A3
BnaGTR2C1
BnaGTR2C2
BnaGTR2C3

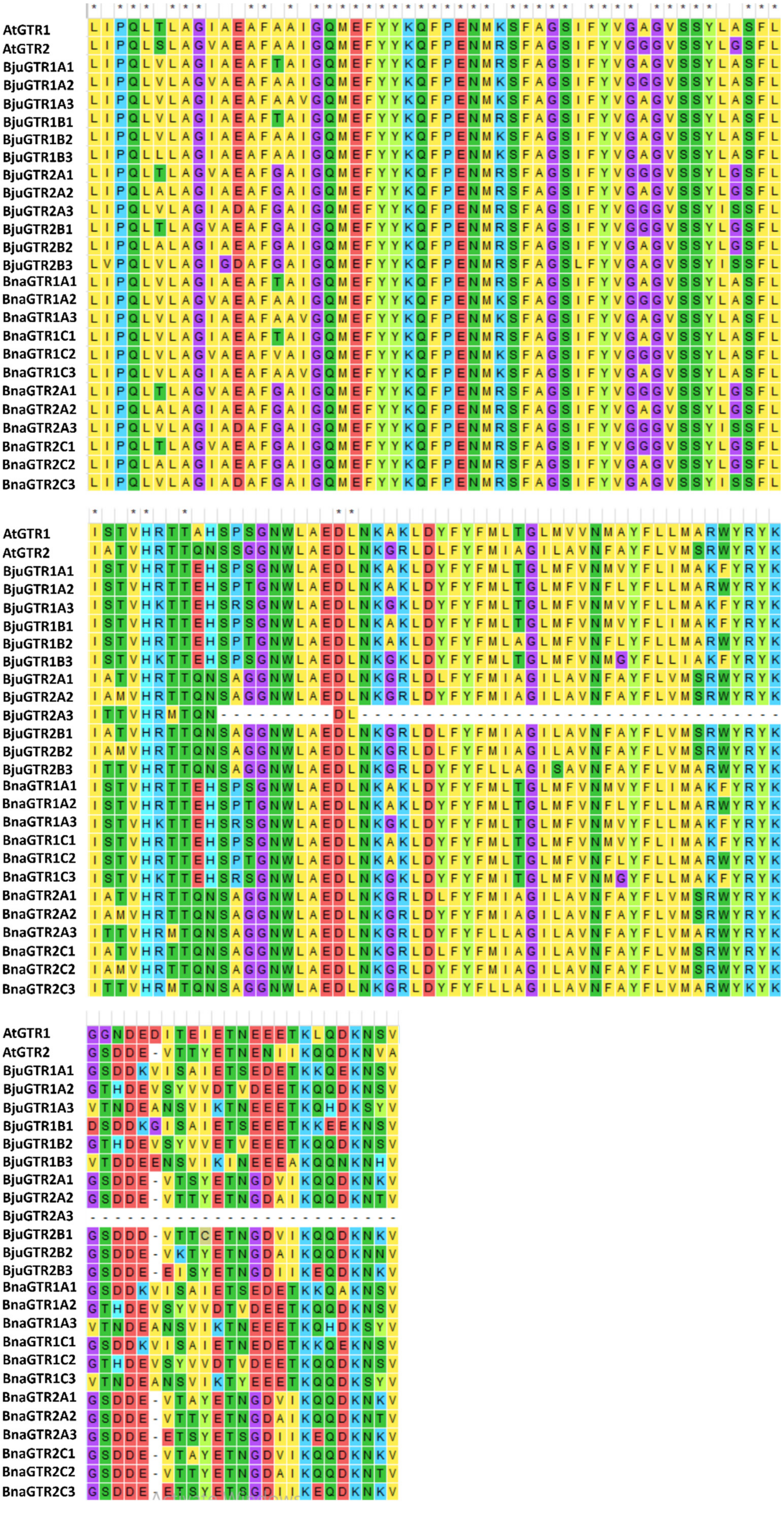
AtGTR1
AtGTR2
BjuGTR1A1
BjuGTR1A2
BjuGTR1A3
BjuGTR1B1
BjuGTR1B2
BjuGTR1B3
BjuGTR2A1
BjuGTR2A2
BjuGTR2A3
BjuGTR2B1
BjuGTR2B2
BjuGTR2B3
BnaGTR1A1
BnaGTR1A2
BnaGTR1A3
BnaGTR1C1
BnaGTR1C2
BnaGTR1C3
BnaGTR2A1
BnaGTR2A2
BnaGTR2A3
BnaGTR2C1
BnaGTR2C2
BnaGTR2C3

Figure S1: Multiple sequence alignment of GTR proteins from Arabidopsis*, B. juncea* and *B. napus.* Multiple sequence alignment was performed by submitting the protein sequences into online available MUSCLE alignment tool using default settings. Different conserved amino acids are represented by different color codes in the picture. The accession number of the query proteins are given as under: AtGTR1 (AT3G47960.1), BnaGTR1A1 (A06p19700.1_BnaDAR), BnaGTR1A2 (A06p27390.1_BnaDAR), BnaGTR1A3 (A01p25730.1_BnaDAR), BnaGTR1C1 (C03p72210.1_BnaDAR), BnaGTR1C2 (C03p66900.1_BnaDAR), BnaGTR1C3(C01p32480.1_BnaDAR), BjuGTR1A1 (BjuVA06G19500), BjuGTR1A2 (BjuVA06G26150), BjuGTR1A3 (BjuVA01G26580), BjuGTR1B1 (BjuVB08G47510), BjuGTR1B2 (BjuVB06G23560), BjuGTR1B3 (BjuVB05G42900), BnaGTR2A1 (A02p42460.1_BnaDAR), BnaGTR2A2 (A06p29420.1_BnaDAR), BnaGTR2A3 (A09p07810.1_BnaDAR), BnaGTR2C1 (C02p62380.1_BnaDAR), BnaGTR2C2 (C03p64110.1_BnaDAR), BnaGTR2C3 (C09p08780.1_BnaDAR), BjuGTR2A1 (BjuVA02G47610), BjuGTR2A2 (BjuVA06G28410), BjuGTR2A3 (BjuVA09G07610), BjuGTR2B1 (BjuVB04G01960), BjuGTR2B2 (BjuVB06G11810), BjuGTR2B3 (BjuVB07G18470).

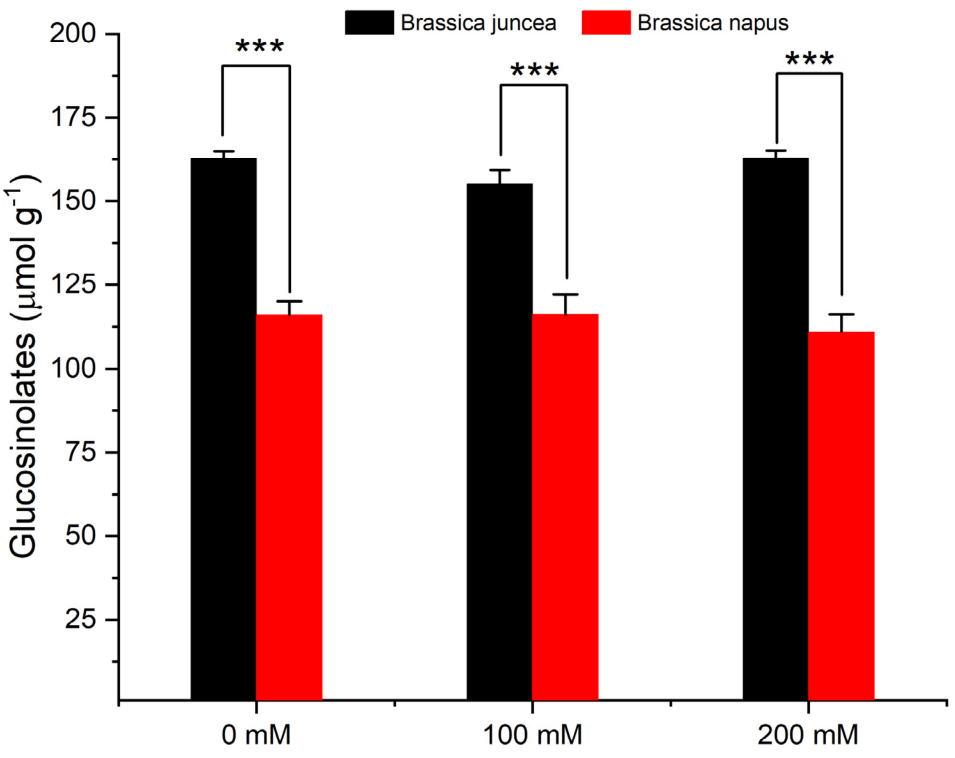

Figure S2: Concentration of overall glucosinolates content in seed. Plants of *B. juncea* (blue lines) and *B. napus* (orange) were grown under 0, 100 and 200 mM NaCl concentrations. Values represent means ± SD from three biological replicates per treatment. Data points represent the means ± SD of three biological replicates. Significant differences are shown with different asterisks (Tukey's HSD, p = 0.05).) Significant codes: ***, **, * at p = 0.0001, 0.001 and 0.01, respectively.)
